# Crops exposed to extreme conditions: perspectives of gene editing to improve stress tolerance

**DOI:** 10.1007/s00299-026-03891-w

**Published:** 2026-06-26

**Authors:** László Szabados, Kamal Kant, Sahilu Rabilu, Afreen Rashid, Laura Zsigmond

**Affiliations:** 1 Institute of Plant Biology, HUN-REN Biological Research Centre, Szeged, Hungary; 2 Department of Plant Biology, University of Szeged, Szeged, Hungary; 3 Ahmadu Bello University, Zaria, Nigeria

**Keywords:** Drought, Salinity, Cold tolerance, Gene editing, CRISPR/Cas9, Crop improvement

## Abstract

Extreme environmental conditions, such as drought, soil salinity, and extreme temperatures, seriously limit agricultural production. Traditional breeding can produce more tolerant cultivars, but has limitations due to the multigene-encoded traits and long time needed to generate improved varieties. Gene editing can introduce single or multiple point mutations, generate microinsertion/deletions (InDel), modify selected bases or DNA fragments, and modulate gene expression, producing novel alleles resembling natural variability. In this review, we cover the strategies and perspectives for editing nuclear or organelle-encoded genes to enhance tolerance to extreme environmental conditions, such as drought, salinity, cold, and heavy metals. A list of 73 arabidopsis genes is assembled, whose function in abiotic stress responses has been demonstrated with insertion or gene-edited mutants. A collection of 97 gene-edited mutants in crops with tolerance to one or more abiotic stresses was compiled. The power of multiplex editing is demonstrated to target multigene-encoded traits related to stress tolerance. Special attention is paid to encouraging results of 20 gene editing programs in various crops, which confirmed improved tolerance in field conditions, demonstrating the feasibility to incorporate this powerful technology into plant breeding. As gene editing generates molecular changes similar to natural polymorphism, legal conditions adopting NGT-friendly frameworks are expected to facilitate commercialization of such engineered varieties in most countries.

**Supplementary Information:**

The online version contains supplementary material available at 10.1007/s00299-026-03891-w.

## Introduction

Crop production is increasingly threatened by extreme and unpredictable environmental conditions, with drought being one of the most damaging abiotic stresses affecting global agriculture. Drought reduces growth, productivity, and yield stability across nearly all major crop species (Ashraf et al. 2021). Response to extreme environmental conditions depends on multiple levels, including cellular, anatomical, morphological, physiological, and molecular levels and is controlled by complex, multigene-encoded regulatory mechanisms. Potential targets in plant stress responses have been identified and thoroughly reviewed earlier (Mittler and Blumwald 2010; Bowerman et al. 2023). At least six signal transduction networks are implicated in the control of tolerance to drought, salinity, or cold (Uno et al. 2000; Fowler and Thomashow 2002; Qiu et al. 2002; Shinozaki and Yamaguchi-Shinozaki 2007; Vlad et al. 2009; Catalá et al. 2011; Zhu 2016; Kim et al. 2024). Improvement of tolerance to such abiotic factors by traditional breeding is usually hindered by the polygenic nature of these traits and the limited available genetic variation. As a consequence of complex regulation, genetic modification (GM) of a single gene usually provides only minor improvements, sometimes accompanied by unintended pleiotropic effects. Due to such constraints, commercially relevant GM crops have been generated with more success with improved resistance against biotic stresses including herbivory insects, pathogenic fungi, or viruses, leading to reduced pesticide use and increased yields demonstrating the potential of genetic engineering in crop improvement (Klümper and Qaim 2014; Yu et al. 2022). Introducing selected genes with particular regulatory or protecting functions could, however, improve plant tolerance to dehydration, salinity, or other abiotic stresses, as demonstrated in controlled conditions, which, however, often failed in the field (Cominelli and Tonelli 2010; Peleg et al. 2012). As a promising exception, maize plants engineered to express bacterial RNA chaperone proteins (CspB) showed improved drought tolerance under both greenhouse and field conditions (Castiglioni et al. 2008). This work contributed to the development of the drought-tolerant GM maize line MON87460 (DroughtGard®), which has been released in several countries in hybrid backgrounds with stacked traits and demonstrated yield advantages under water-limited environments (Monsanto 2012; Nemali et al. 2015; Oyekunle et al. 2023). Several genes have been identified by the company Corteva Agriscience which could enhance yield and drought tolerance of transgenic maize in field conditions (Simmons et al. 2021). These examples demonstrate that genetic modification of key regulatory genes can indeed improve multigene-controlled traits such as tolerance to abiotic stresses. Gene editing offers novel possibilities to modify single or multiple stress targets without integration of large foreign DNAs and engineer complex, multigene-encoded traits which control yield stability in a changing environment (Bowerman et al. 2023; Chavhan et al. 2025). Here we summarize experiments and studies as well as the conceptual and practical strategies offered by genome editing to improve tolerance to extreme environmental conditions, particularly to drought, cold, heavy metals and salinity, focusing on the last 15 years. Reports on edited genes in arabidopsis and various crop plants with relevance to abiotic stress tolerance are listed in supplemental tables. Studies which could demonstrate the beneficial effects of genome editing on soil-grown model or crop plants are emphasized.

## Gene editing in plants

Natural diversity was the main source of genetic variability for traditional crop improvement. Genetic variability in commercial crops is however limited; therefore, alternative gene sources are needed to increase the gene pool for further improvement. Use of wild species for introgression of useful traits is a possibility, which is however limited by incompatibility problems and lengthy selection. Generation of mutants with irradiation or chemical mutagens produces random mutations requiring subsequent selection of the desirable trait followed by multiple backcrosses to fix the new allele (Slewinski et al. 2025). With the advance of direct editing of endogenous genes, powerful tools became available for plant research and breeding purposes. Growing list of edited genes in model and crop plants indicates the potential to use this technology in stress-related discovery research as well as in biotechnological applications aiming improvement of stress resilience of crops (see Supplementary data).

Genome editing employs special nucleases to target particular DNA sequences in the genome, and generate double-stranded breaks (DSBs): Zinc Finger Nucleases (ZFNs), Transcription Activator-Like Effector Nucleases (TALENs), and Clustered Regularly Interspaced Short Palindromic Repeats System linked to Cas nuclease (CRISPR/Cas). Features and uses of these editing tools have extensively been reviewed (Beurdeley et al. 2013; Feng et al. 2013; Gaj et al. 2013; Osakabe et al. 2016; Wada et al. 2020; Son and Park 2022; Wang and Doudna 2023) and will only be briefly summarized below. Most gene editing programs employ *Agrobacterium*-mediated or biolistic genetic transformation of the programmable nuclease constructs into the genome of the host plant. Genetic transformation however can be limited in recalcitrant crops, which can be enhanced by nanoparticles or virus-mediated delivery (Mittler and Blumwald 2010; Demirer et al. 2021; Li et al. 2024a; Tuncel et al. 2025). Alternatively, transient expression of preassembled Cas9–gRNA ribonucleoprotein complexes (RNPs) provides a DNA-free technology to avoid transgene integration and associated regulatory constraints (Woo et al. 2015; Liang et al. 2017; Wada et al. 2020; Gong et al. 2021). Commercial services are now available for GM-free gene editing of a number of crops (https://www.hudsonriver.bio).

Among the available technologies, the CRISPR/Cas system is the most widely used gene editing approach, which can create point mutations, frameshifts, small insertions, or deletions (InDels) (Jinek et al. 2012; Barrangou 2012; Zetsche et al. 2015; Yan et al. 2019; Wada et al. 2020). The CRISPR/Cas9 system has been used to produce mutations, generate allelic variants for plant research and biotechnology (Feng et al. 2013; Nadakuduti and Enciso-Rodríguez 2021; Wang and Doudna 2023; Li et al. 2024a; Nascimento et al. 2023; Tuncel et al. 2025). Promoter editing can enhance or reduce gene expression, resulting in gain- or loss-of function phenotypes, respectively. Such approach was used in rice to generate novel alleles of the *Waxy* gene and to improve grain quality (Huang et al. 2020). CRISPR/Cas9-mediated mutagenesis of *ZmTCP14* created knockout maize lines with improved drought tolerance, demonstrating the utility of this technology for stress biotechnology (Jiao et al. 2023). CRISPR/Cas technology may generate unintended, off-target mutations in the plant genome. Such undesirable side effects can be revealed by whole genome sequencing and their frequency reduced by high-fidelity nucleases, such as eSpCas9(1.1), SpCas9-HF2, HypaCas9 CPF1, or Cas-SF01^HiFi^ (Xu et al. 2019; Khan et al. 2021; Duan et al. 2024). Interestingly, use of ribonucleotide protein complexes (RNPs) generated much lower off-target mutations in wheat when compared to editing with DNA-based CRISPR/Cas9 (Liang et al. 2017).

More recent techniques expand the range of applications by enabling more precise gene editing for genome modifications without introducing double-strand breaks (Li et al. 2024a; Tuncel et al. 2025; Zhao et al. 2025). Base editing generates targeted nucleotide conversions creating point mutations, and can produce both loss- and gain-of function alleles in both model and crop plants (Shimatani et al. 2017; Chen et al. 2017; Kim 2018; Li et al. 2018; Tong et al. 2023; He et al. 2024). Prime editing introduces small DNA substitutions or indels into target DNA employing modified nCas9 fused to a particular reverse transcriptase (RT) and special prime editing gRNA (pegRNA) (Anzalone et al. 2019; Liu et al. 2021). The PrimeRoot approach can insert gene regulatory elements or large gene constructs into plant genomes (Sun et al. 2024). Homology-directed repair (HDR) and homologous recombination (HR) enable targeted insertions, replacements, and point mutations by exchanging native sequences with donor DNA fragments, although their efficiency remains limited in plants (Paszkowski et al. 1988; Hoshijima et al. 2016; Rozov et al. 2019). Frequency of HR can be improved with egg cell-specific expression of the SaCas9 nuclease or using Cas12a or modified Mb2Cas12a endonucleases which could increase frequency by one to two magnitudes (Wolter and Puchta 2019; Zhang et al. 2021). These results suggest that optimized gene editing can be a promising tool for targeted mutagenesis in plants.

Multiplex gene editing employs several guide RNAs for simultaneous targeting and modification of various genes such as members of a gene family or genes which control complex pathways (Li et al. 2013; Xie et al. 2015; Zhang et al. 2016; Wang et al. 2017; Armario-Najera et al. 2019). Efficiency of multiplex editing can benefit from the use of high-fidelity endonucleases such as Cas12a with improved activities (Wolter and Puchta 2019; Zhang et al. 2021). Multiple gene mutations have been generated in a number of plants including rice, wheat, maize, tomato, tobacco, and arabidopsis, demonstrating the potential of this technology (Wang et al. 2018; Armario-Najera et al. 2019; Abdelrahman et al. 2021; Stuttmann et al. 2021). Multiplex editing is a particularly valuable tool to engineer polygenic traits which usually determine tolerance to abiotic stresses in crops with large, polyploid genomes (Wang et al. 2018; Abdelrahman et al. 2021; Nascimento et al. 2023; Ni et al. 2023, see Table 1). In soybean, the quintuple *gmaitr* mutant displayed ABA hypersensitivity and increased salt tolerance which could be confirmed in field conditions (Wang et al. 2021a). Multiplex base or prime editing can simultaneously modify several genes facilitating the engineering complex traits (Shimatani et al. 2017; Abdelrahman et al. 2021; Ni et al. 2023). This technology was used to create herbicide-resistant *OsALS* alleles in rice (Fan et al. 2024). These approaches provide powerful tools for precise genome modification to modify regulatory processes associated with responses to environmental stresses (Nadakuduti and Enciso-Rodríguez 2021; Li et al. 2024a; Chavhan et al. 2025). Examples of multiplex gene editing to improve abiotic stress tolerance in plants are compiled in Table 1.

**Table 1:** Multiplex gene editing used to improve tolerance to extreme environmental conditions

Plant	Genes	Technology	Mutation	Protein	Function	Category	Reference
Arabidopsis	AITR1, 2, 3, 4, 5, 6	CRISPR/Cas9	6 × KO	transcription repressor	ABA signaling, ROS homeostasis	drought	Chen et al 2021a, b, Tian et al 2017
Rice	OsLASPO, OsQS	CRISPR/Cas9	2 × KUp, OX	L-aspartate oxidase, quinolinate synthase	NAD + biosynthesis	development, stress response	Yao et al 2024
Rice	OsPIN5b, GS3, OsMYB30	CRISPR/Cas9	3 × KO	Multiple (TF, signaling)	hormone, transcription	cold, yield	Zeng et al 2020
Rice	OsPSBS1	CRISPR/Cas9	KUp, OX	PSII subunit S	photosynthesis	osmotic	Pattel-Tupper et al. 2024
Rice	OsSRL1, OsSRL2	CRISPR/Cas9	2 × KO	glycosylphosphatidylinositol-anchored protein	leaf rolling, ROS-scavenging	drought	Liao et al 2019
Rice	OsWRKY5	CRISPR/Cas9	2 × KO	transcription factor, WRKY	transcription regulation	drought	Lim et al. 2022
Rice SR86	13 genes	CRISPR/Cas9	13 × mutations	multiple functions (enzymes, TFs, signaling	plant architecture, photoperiod, seed development, etc	salt	Hao et al 2025
Soybean	GmAITR2, 3, 4, 5, 6	CRISPR/Cas9	2x, 5 × KO	transcription repressor	ABA signaling	salt	Wang et al 2021a, b, c
Soybean	GmLHY1a, 1b, 2a, 2b	CRISPR/Cas9	4 × KO	transcription factor	Circadian rhythm	drought	Wang et al 2020
Tobacco	NtAITR1, 2, 3, 5, 6	CRISPR/Cas9	5 × KO	transcription repressor	ABA signaling, ROS homeostasis	drought	Li et al 2022a, b
Tomato	SlHyPRP1	CRISPR/Cas9	2 × KO	hybrid proline-rich protein	cell wall integrity	salt, pathogens	Tran et al 2021, Tran et al. 2023
Tomato	SlHyPRP1, SlDEA1	CRISPR/Cas9	2 × KO	hybrid proline-rich protein, 8CM protein	cell wall integrity	salt, pathogen	Saikia et al 2024
Wheat	TaCYP706B, TaCYP707A	CRISPR/Cas9, dCas9	2 × KD, KO	cytochrome P-450 monooxygenases	ABA catabolism	drought	Li et al 2025
Wheat	TaSal1, 6 genes	CRISPR/Cas9	6 × KO	3'(2'), 5'-bisphosphate nucleotidase	PAP signaling	drought	Mohr et al 2022, Abdallah et al 2025

CRISPRa and CRISPRi systems employ catalytically inactive dCas9 fused to gene activation or repression domains, which can induce or silence transcription (Gilbert et al. 2013; Park et al. 2017). Multiplex gene activation systems can simultaneously induce several genes (Lowder et al. 2018). CRISPR-mediated gene regulation can generate gain or loss-of-function alleles and provide additional tools for crop improvement (Park et al. 2017; Khan et al. 2025).

Editing of organellar genomes became possible with the development of efficient transformation systems of chloroplasts and mitochondria (Yu et al. 2017, 2020; Occhialini et al. 2021; Thagun et al. 2019, 2024). While CRISPR/Cas-based approaches are generally not suitable for organellar genome editing in plants, TALEN-based systems have been successfully employed to generate point mutations and deletions in mitochondrial and plastidic genes (Gammage et al. 2018; Kang et al. 2021; Son and Park 2022; Maliga 2022; Arimura and Nakazato 2024). The mitoTALEN system (mitochondria-targeting TALEN) allowed efficient editing of mitochondrial genomes in various crops and correct mutations which were responsible for cytoplasmic male sterility (Kazama et al. 2019; Arimura 2022; Kuwabara et al. 2022; Maliga 2022; Arimura and Nakazato 2024; Forner 2025). TALEN-linked base editing included TALE-linked adenine deaminases (TALEDs) or deaminase toxin A-derived cytosine base editors (DdCBEs) which could generate point mutations in plastidic and mitochondrial DNA (Kang et al. 2021; Mok et al. 2022, 2024; Zhang and Boch 2024). TALEDs were used with success to mutate chloroplast *psbA* gene and generate herbicide resistance (Mok et al. 2024). Base editing in the mitochondrial genome was achieved with mitochondria-targeting TALEN-based cytidine deaminase (mitoTALECD), targeting the *OTP87* gene which is implicated in editing the mitochondrial *nad7* and *atp1* transcripts (Nakazato et al. 2022). Gene editing of mitochondria and chloroplasts facilitates targeted mutagenesis of organellar genes, many of them implicated in environmental stress responses (Arimura and Nakazato 2024; Forner 2025).

## Gene editing in abiotic stress responses

### Background of drought tolerance

**Fig. 1:**
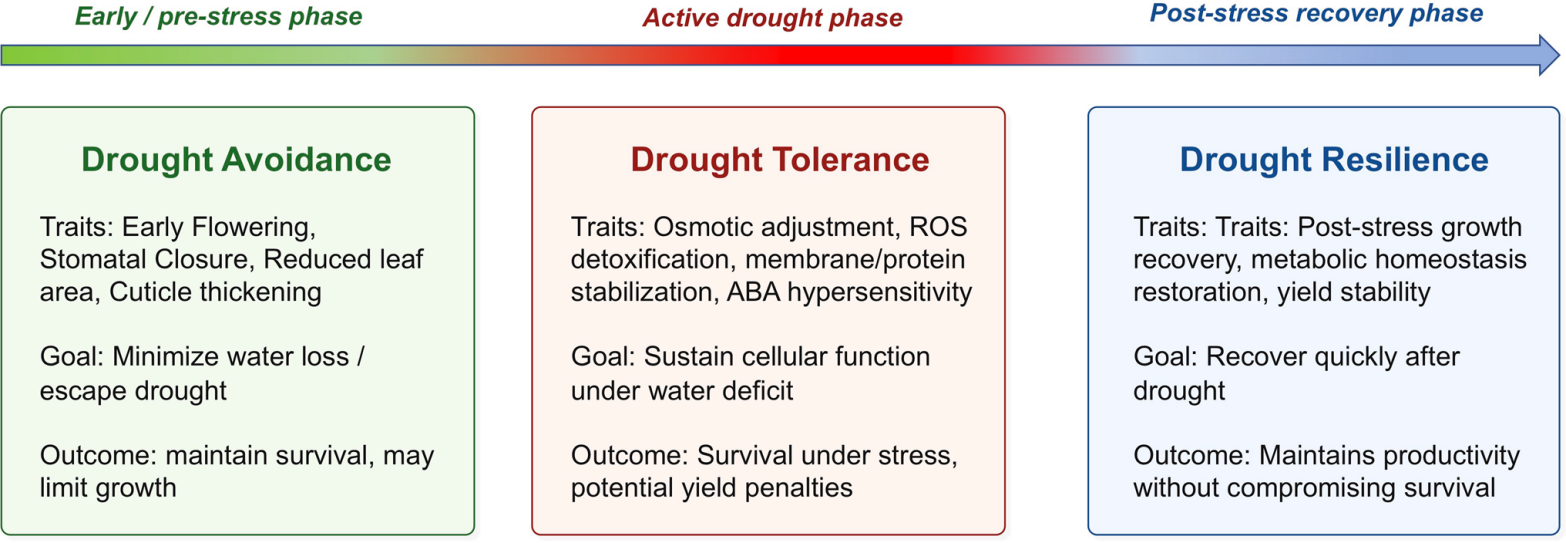
Plant responses to drought. Strategies using drought avoidance, tolerance and resilience. Drought avoidance is mainly based on developmental adaptation, trying to escape or reduce the effect of water depletion. Tolerance traits are key mechanisms on the cellular, metabolic and molecular level, which alleviate the harmful effects of dehydration. Capacity for recovery from water-depleted conditions is key for plant survival in an environment with drought periods

Plants respond to drought through several strategies, such as drought avoidance, drought tolerance, and drought resilience, each reflecting particular developmental and physiological priorities (Fig. 1, Chaves et al. 2003). Drought avoidance encompasses traits that minimize water loss or optimize water capture, such as early flowering, leaf rolling, reduced leaf area, stomatal regulation, and cuticular adjustments, allowing plants to escape or endure transient water scarcity (Chaves et al. 2003; Shavrukov et al. 2017; Ilyas et al. 2021). Drought tolerance refers to cellular- and tissue-level mechanisms that sustain function under water deficit, including osmotic adjustment, detoxification of reactive oxygen species (ROS), stabilization of membranes and proteins. Drought resilience emphasizes post-stress recovery, encompassing the plant’s ability to resume growth, maintain yield, and restore metabolic homeostasis after rehydration (Fang and Xiong 2015; Bandurska 2022). Distinguishing these strategies is critical for identification of genome-editable targets. While enhancing tolerance mechanisms may improve survival under stress, it can inadvertently reduce growth or yield in standard conditions, whereas targeting avoidance or resilience traits can balance survival with productivity (Fig. 1). Improvement of drought tolerance is one of the key issues associated with agricultural problems in changing climate. Adaptation to water-restricted environments is however determined by a number of genes which control water retention, phytohormone content and signals, generation and scavenging of reactive oxygen species, calcium and ROS-related signals, metabolic changes. Several interacting regulatory networks control responses to drought and other stresses, including ABA-dependent and independent signaling pathways (Kim et al. 2024). Such multigene-encoded quantitative characters are not easy to handle and identification of key regulators of such complex processes is a key aspect to design appropriate strategies for gene editing.

### ABA metabolism and signaling

Abscisic acid (ABA) is the principal plant hormone which controls responses to environmental changes leading to dehydration: drought and high soil salinity. ABA regulates seed dehydration, germination, responses to water depletion, including closure of stomata aperture, water use efficiency (WUE), changes in growth and development, and mediates the induction of a large number of stress-responsive genes (Chen et al. 2020; Finkelstein 2013). Most important aspects of ABA perception and regulation have already been identified in the model plant *Arabidopsis thaliana,* and the key signaling pathways characterized. ABA-dependent molecular and physiological processes are controlled by complex interactions of positive and negative regulators which execute either fast responses which do not require changes in gene expression or slower, more extended adaptation, with large-scale changes in transcript profiles (Finkelstein 2013; Yoshida et al. 2019). ABA signaling pathways are quite conserved throughout the plant kingdom, and the knowledge obtained on the arabidopsis model can be easily adapted to most crops (Negin and Moshelion 2016). Targeted mutagenesis of key ABA regulatory genes can therefore be an attractive strategy to improve drought and salt tolerance.

ABA levels are determined by conserved biosynthetic and catabolic pathways. First steps of biosynthesis are localized in the chloroplasts, while the last and rate-limiting reactions take place in the cytosol. The stress-induced *NCED3* mediates the rate-limiting reaction in ABA biosynthesis and controls ABA levels. ABA catabolism is mediated and regulated by P-450 type monooxygenases (Finkelstein 2013). Enhanced ABA content can promote stress responses and increase tolerance to dehydration in plants but can restrict growth and affect seed germination. ABA biosynthesis has been modulated in rice by engineering *OsVDE*, a lipocalin-like protein which downregulates early steps of ABA biosynthesis in chloroplasts. CRISPR/Cas9 editing produced mutants with elevated ABA levels, faster stomatal closure and had higher survival rates in salt-stressed conditions. Salt tolerance of the *osvde* mutant was confirmed in saline paddy fields also (Table 2). Plants were however dwarf and had lower seed setting rates, questioning the agricultural utility of such high ABA lines (Wang et al. 2021b). Lateral organ boundaries domain (LBD) proteins are plant-specific transcription factors which are known to control organ development. *ZmLBD5* of maize was reported to regulate ABA and GA biosynthesis and as a consequence, modulate growth and molecular responses to water deficit. CRISPR/Cas9-derived knockout maize plants were dwarf but had enhanced tolerance to water deficit with higher grain yield in field conditions (Table 2, Feng et al. 2022). As negative regulators of drought tolerance, genes related to *ZmLBD5* can be potential targets for targeted mutagenesis.

**Table 2:** Gene-edited crops with stress tolerance traits tested and confirmed in field conditions

Plant	Gene	Technology	Mutation	Protein	Function	Category	Reference
Maize	ZmARGOS8	CRISPR/Cas9	K-up	Auxin Regulated Gene in Organ Size	ethylene response	drought	Shi et al. 2017
Maize	ZmGA20ox3	CRISPR/Cas9	KO	GA20-oxidase	GA biosynthesis	drought	Liu et al 2023b
maize	ZmLBD5	CRISPR/Cas9	KO	transcription factor, LBD	GA, ABA synthesis	drought	Feng et al 2022
Maize	ZmTCP14	CRISPR/Cas9	KO	Transcription factor, TCP	ROS control	drought	Jiao et al 2023
Rapeseed	BnFTA	RNAi	KD	Farnesyltransferase	ABA signal	drought	Wang et al 2009
Rice	OsAAA-1	CRISPR/Cas9, RNAi	KO, KD	mitochondrial AAA ATPase	electron transport	drought	Lu et al 2020
Rice	OsCKX2	CRISPR/Cas9	KO	cytokinin oxidase	cytokinin catabolism	drought, yield	Rashid et al 2024
Rice	OsCKX2	CRISPR/Cas12a	KO	cytokinin oxidase	cytokinin catabolism	drought, yield	Solanki et al 2026
Rice	OsDST	CRISPR/Cas9	KO	transcription factor, ZnF	Nitrogen metabolism	drought	Han et al 2022
Rice	OsHAK1	CRISPR/Cas9	KO	K + transporter	K + , Cs + transport	heavy metal/Cs	Nieves-Cordones et al 2017
Rice	OsLCD	CRISPR/Cas9	KO	Unknown	Cd2 + transport	heavy metal/Cd	Chen et al. 2023
Rice	OsLCT1	CRISPR/Cas9	KO	Low affinity cation transporter	Cd2 + transport	heavy metal/Cd	Songmei et al 2019
Rice	OsNRAMP5	CRISPR/Cas9	KO	Cd and Mn transporter	Cd2 + , Mn2 + transport	heavy metal/Cd	Songmei et al 2019
Rice	OsPYL9	CRISPR/Cas9	KO	ABA rceptor, PYL	ABA signaling	drought	Usman et al 2020
Rice	OsRR22	CRISPR/Cas9	KO	transcription factor, B-type RR	cytokinin response	drought, salt	Liu et al 2023a, b
Rice	OsVDE	CRISPR/Cas9	KD	Violaxanthin deoxidase	ABA biosynthesis	salt	Wang et al 2021a, b, c
Rice	unknown	CRISPR/Cas9	KO	transcription repressor	stress response	salt, drought	Priyadarshini 2025
Rice SR86 line	13 agronomic characters	CRISPR/Cas9	13 × KO, K-up	13 proteins	Development, photoperiod, seed set, quality	salt, yield	Hao et al. 2025
Soybean	GmAITR2,3,4,5,6	CRISPR/Cas9	2x, 5 × KO	transcription repressor	ABA signaling	salt	Wang et al 2021a, b, c
Tomato	SlGID1a	CRISPR/Cas9	KO	GA receptor, GID1a	GA signaling	drought	Illouz-Eliaz et al 2020

*Kup* knock-up, *KO* knock-out, *KD* knock-down

Besides biosynthesis, ABA catabolism is an important regulatory step which determines ABA turnover (Finkelstein 2013). CYP707A hydrolases control ABA catabolism by hydroxylation of ABA, which is a key step in ABA degradation (Saito et al. 2004). ABA catabolism is suppressed in the arabidopsis *cyp707a3* T-DNA insertion mutant, which accumulates ABA to high levels leading to reduced respiration and enhanced ABA-dependent gene expression. High ABA levels in such mutants promote ABA signals, leading to superior tolerance to dehydration (Umezawa et al. 2006). Suppression of ABA catabolism has been achieved in wheat by CRISPR/Cas9 editing of *TaCYP706B* and *TaCYP707A* genes. Silencing of *TaCYP707A* could be achieved by modulation of histone methylation at that locus. Blocking the Jumonji (JMJ) demethylase binding site of *TaCYP707A* gene with dead Cas9 (dCas9) increased the abundance of the repressive H3K27me3 marks, reducing transcript levels. Compromised ABA catabolism in these mutants leads to elevated ABA content, improved tolerance to water depletion, but reduced fertility (Li et al. 2025). In rice, *OsABA8ox2* encodes abscisic acid 8′-hydroxylase enzyme, which controls ABA degradation. Knockout mutation of *OsABA8ox2* could be generated by CRISPR/Cas9 editing, leading to increased ABA content, which limited stomatal conductance and promoted vertical root growth. Changes in root architecture and transpiration contributed significantly to drought tolerance, confirmed by enhanced survival rates after water deprivation (Zhang et al. 2020). *OsABA8ox2* overexpressing plants had opposite phenotype, confirming the regulatory function of this gene on ABA levels in rice. Large-scale transcript profiling and gene mapping have identified Zm*ABH2* gene in maize which encodes the abscisic acid 8-hydroxylase (ABAox), catalysing the first step in ABA degradation and functions as a negative regulator of drought tolerance. CRISPR/Cas9-generated mutation in the Zm*ABH2* gene compromised ABA catabolism, promoted ABA accumulation and stomatal closure in response to dehydration. The *ZmABH2* mutant had improved water retention and plant survival in water-restricted conditions (Liu et al. 2020a). These data demonstrate that engineering ABA metabolism in crops by editing regulatory or metabolic genes can be an efficient way to modulate ABA accumulation and improve drought tolerance. High ABA content can however have undesired consequences, such as blocked seed germination, reduced plant growth, fertility, and yield. Such negative effects can probably be alleviated by developmental, cell-specific (guard cells) or stress-dependent control of ABA turnover.

Alteration of ABA signal transduction allows more precise targeting of stress-related functions. ABA is perceived by the PYL/PP2C receptor complex, activating the SnRK2-type OST1 kinase, inducing ion transporters to change turgor of guard cells which is influenced also by calcium and ROS signals (Finkelstein 2013; Rodrigues and Shan 2022). A number of genes regulate stomata closure and can be considered for engineering to control evaporation in water-restricted environment. Activating ABA signaling via overexpression of RCAR/PYL-type ABA receptors reduced stomatal conductance and transpiration, enhanced WUE in water-restricted conditions (Yang et al. 2016). Multiplex CRISPR/Cas9 editing generated sextuple knockouts of ABA receptor genes in arabidopsis (Zhang et al. 2016). ABA hypersensitivity however can be associated with growth restriction which limits its utility in crop improvement. Targeting ABA hypersensitivity to guard cells by cell-specific overexpression of ABA receptors could however improve WUE without growth penalty (Liu et al. 2025a). Rice has 13 PYL-type ABA receptors, some of which have important functions in ABA signaling. Overexpression of *OsPYL3* and *OsPYL9* in rice was found to enhance ABA response during germination and improved drought and cold tolerance. (Tian et al. 2017). CRISPR/Cas9-mediated mutagenesis of *OsPYL9* generated a knockout mutant with elevated ABA content. The *OsPYL9* mutant had reduced stomatal conductance, better antioxidant activity and better recovery rates after water deficiency. Improved drought tolerance has been observed in open field conditions also, with better grain yields in both well-watered and drought conditions (Table 2, Usman et al. 2020). In connection with ABA perception, ROS-dependent Ca^2+^ is an important component of ABA signaling in guard cells. The calcium sensor CBL1/9-CIPK1 phosphorylates PYL/RCAR ABA receptors and negatively regulates downstream signals. Such inhibitory effect is abolished in the *cbl1/9* and *cipk1* mutants, which are hypersensitive to ABA. Faster stomatal closure of the mutants in water-deficient environment reduced evaporation and increased water retention, leading to enhanced tolerance to drought (You et al. 2023). OST2 is an ATP-dependent proton pump which regulates stomata closure upon dehydration. CRISPR/Cas9 editing generated dominant negative *OST2* mutant alleles in arabidopsis. The mutant had fast ABA-triggered stomatal closure and reduced transpirational water loss in water-depleted conditions (Osakabe et al. 2016). These results demonstrate that modulation of stomata-specific ABA signals is feasible by gene editing which can improve water retention in water-restricted conditions and enhance drought tolerance without affecting growth and yield.

The ABA hypersensitive *era1-2* mutant was produced by fast neutrons, which deleted the *AT5G40280* gene. In a water-restricted environment, fast stomatal closing reduced transpirational water loss and increased water retention. The *ERA1* gene encodes a protein farnesyltransferase, which was shown to suppress ABA signals in guard cells (Pei et al. 1998; Allen et al. 2002). Heat shock protein 40 (HSP40) is an important target of ERA1 as mutants with deficient farnesylation of HSP40 were found to exhibit ABA hypersensitivity similar to *era1*. Activation of various stress-induced miRNA genes can be controlled by the SPL7 transcription factor, which also depends on HSP40 farnesylation (Barghetti et al. 2017). Among the HSP40/SPL7-controlled microRNAs, miRNA408 is implicated in abiotic stress responses, functioning as a regulatory hub (Ma et al. 2015). In vegetative tissues, the blue copper protein PLANTACYANIN (PCY) was found to be the primary target of microRNA408 (miR408), repressing *PCY* expression. ABA-dependent down-regulation of miR408 allows *PCY* activation, which generates ROS in guard cells, promoting stomatal closure leading to drought tolerance (Yang et al. 2024). These reports demonstrated the feasibility of engineering ABA sensitivity of guard cells by farnesyltransferases, which can promote stomatal closing and reduction of water loss in water-limited conditions. Such strategy was explored in rapeseed by shoot-specific down-regulation of endogenous farnesyltransferase by a stress-inducible gene silencing construct. Engineered lines were tested in open field conditions where they produced 5% to 20% higher yields than wild-type plants in drought condition (Table 2, Wang et al. 2009). Downstream regulators such as miR408 can be targets for genome editing in crops, to boost stomatal closure and improve drought tolerance.

The nuclear mRNA Cap Binding Complex (nCBC) has two subunits which are implicated in ABA sensitivity. The *abh1* (ABA hypersensitive 1) mutant is defective in the gene which encodes the larger CBC subunit, the 80 kDa Cap Binding Protein 1 (CBP80). *abh1* has enhanced ABA-triggered cytosolic calcium accumulation, which amplifies ABA signals augmenting stomatal closure, water retention and reduced wilting in drought conditions (Hugouvieux et al. 2001). The Cap Binding Protein 20 (CBP20) is another subunit of CBC. Similar to *abh1*, the *cbp20* mutant is also hypersensitive to ABA, displays reduced stomatal conductance and tolerance to water deficit (Papp et al. 2004). These data demonstrate that ABA signals are influenced by the mRNA processing CBC complex, and both CBP20 and CBP80 subunits are essential for the proper regulation (Kuhn et al. 2008). CBC is conserved in plants and recent reports confirmed that editing CBP80 genes in crops is a feasible strategy to improve drought tolerance. *StCBP80* genes in a tetraploid commercial potato variety were edited by CRISPR/Cas9 system, generating several mutant alleles. Enhanced tolerance to dehydration was observed in the mutants, accompanied by accelerated stress response at molecular level, and superior biomass and tuber production (Decima Oneto et al. 2025).

PP2C-type protein phosphatases are important components of ABA perception and signal transduction and several members of this family function as negative regulators of ABA signaling (Finkelstein 2013). While overexpression of *PP2CA* confers ABA insensitivity to arabidopsis, T-DNA insertion *pp2ca* mutants are hypersensitive to ABA displaying enhanced stomatal closure. Moreover, *AtPP2C* is downregulated in the *abh1* mutants, while overexpression of this gene can partially complement ABA hypersensitivity of *abh1* (Kuhn et al. 2006). These results suggest that *ABH1* and *PP2C* control partially overlapping ABA signaling pathways.

Stress-responsive gene expression is regulated by ABA-dependent and independent signaling pathways (Shinozaki and Yamaguchi-Shinozaki 2007; Kim et al. 2024). ABA-regulated gene expression is mainly controlled by ZnF, bZIP and MYB-type transcription factors, and is influenced by chromatin structure (Finkelstein 2013). Engineering such regulatory factors by gene editing offers a range of possibilities to influence drought and salt tolerance. The ABA-Induced Transcription Repressor family has six members in arabidopsis (*AITR1-6*) which act as feedback regulators of ABA signaling. ABA-induced activation of *PP2C* and *PYR/PYL/RCAR* genes was reduced in *AITR* overexpressing plants but was enhanced in multiple *aitr* mutants, suggesting that AITR factors are negative regulators of ABA signals (Tian et al. 2017). Individual *aitr* mutants had no influence on ABA sensitivity. Multiple *aitr* mutants could be generated by CRISPR/Cas9 gene editing which displayed higher survival rates than wild-type arabidopsis after water depletion and no growth penalty in standard conditions (Chen et al. 2019, 2021a). Based on the arabidopsis results, multiple *NtAITR* genes of tobacco were mutated by multiplex CRISPR/Cas9 method. The *ntaitr1,2,3,5,6* quintuple mutants recovered water deficiency with higher frequency than wild-type plants (Li et al. 2022a, b). Similar strategy was adopted for soybean, using CRISPR/Cas9 to target six *GmAITR* genes. Double *bmaitr36* and quintuple *gmaitr2,3,4,5,6* soybean mutants were obtained which showed enhanced ABA sensitivity and tolerance to salinity. Salt tolerance could also be confirmed in field experiments (Table 2, Wang et al. 2021a). These reports suggest that simultaneous editing of various members of a redundant gene family such as AITR is feasible by the CRISPR/Cas9 system. Such strategy can be adapted to different species and is suitable to modulate ABA signaling pathways to improve salt or drought tolerance of crop plants.

HAT1 and HAT3 are closely related HD-ZIP transcription factors which are repressors of ABA-dependent drought response. The double *hat1,hat3* mutant was hypersensitive to ABA, had enhanced ABA levels and increased tolerance to water deprivation (Tan et al. 2018). Moreover, HAT1 is a negative regulator of the plastid to nucleus retrograde signaling, an important pathway for transcriptional reprogramming in stress conditions (Zeng et al. 2025). HAT factors can therefore be promising targets for genome editing to promote ABA-dependent drought tolerance. The DREB-type transcription factors are key regulators of drought responses (Shinozaki and Yamaguchi-Shinozaki 2007). *TaDTG6-B* of wheat is associated with drought tolerance. A 26 bp deletion in the coding region (*TaDTG6-B Del574*) generated a gain-of function allele with stronger promoter binding, promoting target gene expression and drought tolerance (Mei et al. 2022). The mutant allele could be introduced into drought sensitive wheat cultivars and improve their drought tolerance. More recently, TaDTG6-B was shown to control *TaPIF1* expression, which activated a range of stress-related target genes implicated in ABA signaling, stomata closure, proline biosynthesis or dehydration protection (Du et al. 2025). Engineering of DREB-type transcription factors can therefore modulate the activity of a complex regulatory module and improve efficiency of drought responses in crops.

DST encodes a zinc finger transcription factor which controls the expression of nitrate reductase (*OsNR1.2*) in rice. Point mutation in the *OsDST* gene reduced stomatal density and enhanced stomatal closure (Huang et al. 2009). Editing of the *OsDST* gene in *indica* rice generated a loss-of-function deletion mutant with similar characteristics (Santosh-Kumar et al. 2020). Both *osdst* mutants had enhanced tolerance to osmotic and salt stress due to improved water retention (Huang et al. 2009; Santosh-Kumar et al. 2020). Role of DST in nitrogen assimilation and stomatal closure as well as in drought tolerance could be confirmed in field experiments (Table 2, Han et al. 2022). The wheat NAC transcription factor *TaNAC071-A* was identified in a GWAS study and shown to determine drought tolerance. A 108-bp MYB TF-binding fragment in the promoter was identified to be responsible for elevated expression and drought tolerance which could be transferred to sensitive cultivars (Mao et al. 2022). Insertion of such cis regulatory elements into promoters of stress-related genes to boost their expression can be an attractive strategy for gene editing.

Protein stability and turnover are an important aspect of posttranscriptional regulation with implications in stress and ABA signaling. E3 ubiquitin ligases determine ubiquitination, which targets proteins for degradation. PKL was shown to interact with the SUMO E3 ligase MMS21, enhancing its stability (Jing et al. 2023). *MMS21* expression is inhibited by high osmotic conditions and ABA. The *MMS21* mutant on the other hand displayed higher survival rates under water-limited conditions. Stomatal closure and activation of a set of stress-responsive genes in the *MMS21* mutant were hypersensitive to ABA and water deficit (Zhang et al. 2013). These data suggest that MMS21 together with PKL and AFL form a regulatory module which can easily be targeted by genome editing to generate mutants in crops with enhanced drought tolerance. The rice *OsPUB7* gene encodes a U-box protein, which was targeted for CRISPR/Cas9 gene editing. The mutants tolerate better water stress and salinity than wild-type plants. Other U-box genes had increased transcript levels in the mutant suggesting that *OsPUB7* is a key component of a regulatory network and therefore is an attractive target for genome editing to improve salt and drought tolerance (Kim et al. 2023).

These examples demonstrate that a number of genes, implicated in ABA metabolism and signaling, can be potential targets of gene editing. Engineering plants for ABA accumulation or hypersensitivity however might have undesired effects on seed germination, seedling growth, plant development or fertility. Such strategies should target regulatory genes with particular tissue specificity or developmental control to avoid undesired negative effects on plant development and fertility. Careful planning and testing are therefore important to design the editing strategy which can enhance tolerance with minimal side effects.

### Plant hormones in drought tolerance

ABA is not the only plant hormone which regulates drought and salt tolerance. Several other stress regulators are known which often interact or interfere with ABA action. Ethylene is known to influence responses to abiotic stresses and modulates ABA signals. The *ARGOS* gene family modulates ethylene sensitivity by affecting ethylene perception in arabidopsis. Overexpression of *ARGOS* genes in transgenic arabidopsis and maize plants reduced ethylene sensitivity and improved their tolerance to water depletion (Shi et al. 2015). ARGOS-type proteins were shown to interact with ethylene receptor complex and thereby downregulate ethylene signaling (Shi et al. 2016). CRISPR/Cas9-mediated homology directed repair (HDR) was used to replace the endogenous promoter of *ZmARGOS8* with stronger GOS2 promoter in maize and boost the transcription of this gene. The *GOS2-ARGOS8* variant lines displayed enhanced tolerance to drought in field conditions producing significantly improved yields without having yield penalty in well-watered conditions (Table 2, Shi et al. 2017).

DELLA proteins are negative regulators of GA signaling, and are known to interact with ABA-related transcription factors to promote expression of ABA-induced genes. Gain-of function mutants of *BnaA6.RGA*, the arabidopsis RGA homolog in rapeseed were created by CRISPR/Cas9 editing. Stomatal closure of the mutants was hypersensitive to ABA, leading to lower water loss and higher survival rates after water depletion. Stress and ABA-induced genes had enhanced transcript levels in these mutants, suggesting that ABA hypersensitivity was not restricted to stomata control, but contributed to general defenses in other rapeseed tissues (Wu et al. 2020). In tomato, the GIBBERELLIN-INSENSITIVE DWARF1a (GID1a) GA receptor was edited by CRISPR/Cas9, producing knockout mutant alleles. Although there are three *GID1* genes in tomato with overlapping function, the *gid1a* mutant had reduced transpiration and better recovery after dehydration. Stronger alleles affected xylem formation and displayed semidwarf growth habit in field condition, with higher harvest index but no difference in drought response (Table 2, Illouz-Eliaz et al. 2020). GA biosynthesis was inhibited in maize knockout mutants of *ZmGA20ox3* gene resulting in low GA accumulation but increased ABA and JA levels. While growth of the mutants was inferior in standard conditions, drought-triggered yield loss was alleviated in field trials (Table 2, Liu et al. 2023b). Suppression of GA biosynthesis by artificial miRNA inhibition of *ZmGA20ox3* and *ZmGA20ox5* genes could also generate semidwarf maize plants, confirming the importance of GA accumulation in control of maize stature and stress response (Paciorek et al. 2022).

Brassinosteroids (BR) regulate plant growth and architecture, and interfere with the action of other hormones including ABA. The NAC-type transcription factor OsNAC016 of rice was shown to regulate BR biosynthesis and development. This transcription factor represents a crosslink between BR and ABA signaling as the gene-edited knockout plants displayed enhanced, while overexpressing transgenics had reduced drought tolerance in a controlled environment. OsNAC016 promoted and repressed the activity of BR and ABA-induced genes, respectively. The gene-edited *osnac016* mutant could withstand water depletion better than wild-type rice plants due to the derepression of ABA-induced genes (Wu et al. 2022).

Cytokinins (CK) are known to regulate cell division, plant development, and senescence and influence stress responses. Cytokinins are catabolized by cytokinin oxidase (CKX), which controls internal CK content. The *OsCKX2* gene was inactivated by CRISPR/Cas9 gene editing in *indica* rice, which increased internal cytokinin content of the mutant. *oscxk2* displayed enhanced tolerance to dehydration through reduced transpiration, improved photosynthesis, and higher antioxidant activity. Field experiments confirmed higher yields in drought conditions (Rashid et al. 2024). *OsCXK2* was knocked out in a commercial Indian variety Samba Mahsuri with CRISPR/Cas12-mediated editing, removing the FAD and cytokinin binding domains of the enzyme. The KAMALA line was selected among the *oscxk2* mutants, as it had the best agronomic performance in field conditions including yield and moderate drought tolerance, leading to its commercial release in India (ICAR-IIR 2025; Solanki et al. 2026).

Interaction of ABA, jasmonate and auxin signaling has already been reported with auxin response factors (ARF) functioning as important regulators in such a module (Sybilska and Daszkowska-Golec 2023). Tomato has 24 *ARF* genes with considerable variation in their expression profile. *SlARF4* was reported to control ABA–auxin interaction and modulate tolerance to water deficit. *SlARF4* antisense silenced lines and CRISPR/Cas9-generated knockout *slarf4* mutants could tolerate dehydration better, had more abundant roots, thick stems, curly leaves, altered stomata morphology, higher antioxidant levels, and reduced oxidative damage. Hundreds of genes were differentially expressed in this mutant including *SlABI5*, a key regulator of ABA signaling, which can be at least partially responsible for the tolerance of the *slarf4* mutant. *Slarf4* mutants had enhanced salt tolerance, which was due to higher ABA content, reduced stomatal conductance and enhanced expression of antioxidant genes (Bouzroud et al. 2020; Chen et al. 2021b). Interactions of ABA with other hormonal regulatory pathways can therefore influence stress responses, and are amenable for engineering with genome editing in crops to improve drought and salt tolerance.

### Metabolic regulation in drought responses

Adverse environmental conditions profoundly affect plant metabolism, which can influence stress tolerance in various ways. ROS are produced in a number of reactions during plant development and as a consequence of abiotic or biotic stresses. Main ROS producing sites are chloroplasts, mitochondria, peroxisomes and apoplast; generation and scavenging of ROS is important to maintain cellular homeostasis. While uncontrolled ROS accumulation is damaging and generates oxidative stresses, ROS are important secondary signals, implicated in regulation of plant development, hormone signaling, plant defenses and adaptation to extreme environmental conditions (Choudhury et al. 2017). WUSCHEL-related homeobox (WOX) transcription factors are key regulators of plant development and were shown to modulate antioxidant responses to environmental stresses. The *SlWOX4* gene in tomato was targeted by CRISPR/Cas9 editing generating a knockout mutant. The *slwox4* mutant had faster stomata closure and reduced water loss than wild-type plants in water-restricted conditions. More efficient antioxidant system of the mutant alleviated oxidative damage which was considered a key aspect of improved drought tolerance (Li et al. 2024b). Pipecoline acid (PIP) is a lysine catabolite which is implicated in SA signaling. *SlALD1* gene encodes L-lysine alpha-aminotransferase, which catalyses the rate-limiting step in tomato PIP biosynthesis. The gene-edited *slald1* knockout mutant had higher antioxidant activity, reduced ROS accumulation and oxidative damage, increased photosynthesis, leading to enhanced tolerance to water stress (Wang et al. 2021c). These results demonstrate that engineering the antioxidant defenses can be a promising target for genome editing to improve drought tolerance of crops.

Transcription factors belonging to the TEOSINTE BRANCHED1/CYCLOIDEA/PROLIFERATING CELL FACTOR (TCP) family regulate plant growth, development and responses to environmental effects. The maize ZmTCP14 factor has been shown to regulate drought tolerance through modulating ROS metabolism. Overexpression of *ZmTCP14* enhanced ROS accumulation and compromised drought responses. Gene-edited *zmtcp14* knockout mutants had a more abundant root system, had reduced ROS levels in water-limited conditions and recovered at higher frequency after re-watering. Enhanced tolerance of *zmtcp14* could be validated in open field also, as they had only minimal yield loss in drought conditions (Table 2, Jiao et al. 2023).

Besides ROS, a number of metabolites with protective or signaling properties have been identified. Trehalose is a disaccharide which accumulates to high concentrations in several extremophile plants during desiccation. Overexpression of trehalose-6-phosphate synthase (TPS1), a key enzyme in its biosynthetic pathway in transgenic plants was shown to promote trehalose accumulation and improve tolerance to water deprivation (Romero et al. 1997). The protecting effect of trehalose accumulation was demonstrated in several transgenic crops, engineered to boost the biosynthetic pathway including rice (Garg et al. 2002; Jang et al. 2003) and potato (Kondrák et al. 2012). Blocking trehalose catabolism could be achieved by editing the substrate-binding domain of trehalase, which resulted in trehalose accumulation. T-DNA insertion *tre1* and edited trehalase knockout arabidopsis mutants displayed similar tolerance to water deprivation, suggesting that the trehalose pathway is a potential target for crops also (Nuñez-Muñoz et al. 2021). Gene expression can be boosted by inserting enhancer sequences into promoters using CRISPR/Cas9 editing. Multiplexed upregulation of L-aspartate oxidase (LASPO) and quinolinate synthase (QS) genes in rice could be achieved by inserting short transcriptional enhancers into gene promoters, enhancing nicotinamide mononucleotide (NMN) levels, a precursor for NAD + biosynthesis (Yao et al. 2024).

Wax layer in the cuticle is an important barrier of evaporation and can influence water retention during water shortages. The ECERIFERUM9 (*CER9*) gene encodes an E3 ubiquitin ligase which is implicated in cuticular wax biosynthesis. The arabidopsis *cer9* insertion mutant was found to have elevated amounts of very-long-chain free fatty acids increasing the cuticle membrane thickness on epidermal cells. During water deficit, *cer9* leaves had delayed wilting, reduced transpiration, improved water use efficiency leading to enhanced tolerance (Lü et al. 2012).

### Salt tolerance

Salinity is the consequence of extreme accumulation of ions, mainly sodium, calcium, or magnesium, accompanied by chlorides, sulfates, or carbonates in the soil. Millions of hectares are affected by salts in arid regions, in zones exposed to seawater or in extensively irrigated areas. Salts affect plant growth in multiple ways. High salt concentration increases osmolarity, preventing efficient water uptake of roots generating osmotic stress. Ions such as sodium or chloride are toxic, affecting metabolic processes through inhibition of enzyme activities. Sodium and chloride can reduce uptake of potassium or nitrate, respectively, leading to nutrient starvation (Munns and Tester 2008). Maintenance of ion homeostasis in plants is a critical aspect of salt tolerance which is controlled by a complex system, composed of membrane sensors, signaling proteins, such as protein kinases and phosphatases, transcriptional regulators, and specific transporters localized in plasma membranes or internal membrane systems (Munns and Tester 2008; Yang and Guo 2018). Uptake and partitioning of Na^+^ ions is an essential adaptive mechanism which is mediated by various classes of ion transporters whose activity is largely determined by protein phosphorylation and controlled by the Salt Overtly Sensitive (SOS) signaling system (Ji et al. 2013; Yang and Guo 2018). The SOS pathway is composed of the EF-hand calcium-binding protein SOS3 and the SOS3-LIKE CALCIUM BINDING PROTEIN8 (SCaBP8), sensing the calcium signal stimulated by salinity. SOS3/SCaBP8 interacts with and activates the serine/threonine protein kinase SOS2, which subsequently phosphorylates the plasma membrane Na + /H + antiporter SOS1. SOS1 transports Na^+^ ions from cytoplasm to apoplast, reducing the intracellular concentration of this toxic ion. The SOS pathway is the principal mechanism to control sodium toxicity and maintain ion homeostasis of plant cells (Hasegawa et al. 2000; Yang and Guo 2018).

Selectivity of ion transport is essential to differentiate between harmful ions, such as Na^+^ or Cs^+^ and essential elements like K^+^. Specificity and activity of the transporters can be altered by gene editing to influence ion homeostasis and improve tolerance to salinity and other toxic metals. The arabidopsis AtHAK5 high-affinity K^+^ transporter was modified by changing phenylalanine 130 to serine (F130S) to improve K^+^ uptake and reduce Na^+^ transport. The mutation led to higher K^+^ content and accumulated less Na^+^ or Cs^+^ ions leading to a certain degree of salt tolerance (Jiménez-Estévez et al. 2024). Mutations of ion transporters of crop plants were also shown to influence ion transport and homeostasis. Genome-wide association study (GWAS) identified variation in the coding sequence of the tomato *SlHAK20* gene, associated with difference in Na^+^ and K^+^ balance and salt sensitivity (Wang et al. 2020).

Activity of ion transporters is controlled by protein phosphorylation, specific signaling and transcription factors having positive or negative effect on their activity. The SnRK2-type protein kinase SOS2 phosphorylates and activates the plasmalemma localized SOS1, and the vacuolar Na^+^/H^+^ antiporter NHX, both of them are important for Na^+^ exclusion from the cytoplasm. On the other hand, some of the calcium-dependent kinases function as negative regulators. The salt-induced OsCIPK9 in rice interacts with the calcium-binding protein OsSOS3 and inhibits the activity of important ion transporters OsKAT1 and OsNHX1. The CRISPR/Cas9-generated *OsCIPK9* knockout line had enhanced expression of key ion transporter genes *KAT1* and *NHX1* and was more tolerant to salinity (Zhou et al. 2023). The B-type response regulator transcription factor OsRR22 is a component of the cytokinin signaling system in rice and is implicated in salt responses. A genetic screen has identified *hst1*, a mutant allele of *OsRR22* gene, with considerably enhanced salt tolerance displayed also in field. The mutant was subsequently used to develop a salt-tolerant commercial rice variety (Takagi et al. 2015). Knockout mutant alleles of *OsRR22* were later generated by CRISPR/Cas9 genome editing in a different rice background and were shown to be resilient to salinity, which could be confirmed in field conditions (Table 2; Zhang et al. 2019; Liu et al. 2023a). Together with the DELLA protein OsSLR1, OsRR22 was found to control the expression of *OsHKT2,1*, a “HIGH-AFFINITY POTASSIUM (K +) TRANSPORTER” gene, responsible for sodium uptake in rice (Liu et al. 2023a). While many transcription factors have been identified which activate salt-responsive genes, factors with negative regulatory function are less known. One of them is *OsbHLH024* in rice which was shown to downregulate numerous target genes implicated in salt tolerance. The knockout *OsbHLH024* mutant generated with CRISPR/Cas9 technology was less sensitive to salinity, had reduced ROS content and oxidative damage, superior photosynthetic activity, more balanced ion content and increased survival in saline conditions. Primary reason of the observed tolerance was the upregulation of key ion transporter genes *OsHKT1;3*, *OsHAK7* and *OsSOS1*, responsible for contained Na^+^ accumulation and improved ion balance (Alam et al. 2022). The HD-ZIP II-type transcription factor SlABIG1 of tomato is another example for negative regulators of salt tolerance. The gene-edited *SlABIG1* mutant had higher chlorophyll content and photosynthesis, reduced ROS accumulation, and oxidative damage. Reduced Na^+^ and enhanced proline content were suggested to be responsible for salt tolerance of this mutant (Ding et al. 2022). These results demonstrate that mutagenesis of certain protein kinases, regulatory proteins and transcription factors can enhance tolerance to salt stress through promoting the activities of ion transporters to maintain ion homeostasis.

Besides ion transporters, salt tolerance is influenced by various metabolic processes. One of them is inositol metabolism, which was engineered by gene editing in rice. Conversion of *myo*-inositol-1,3,4,5,6-penta*kis*phosphate to *myo*-inositol-1,2,3,4,5,6-hexa*kis*phosphate (IP_6_) in rice is catalysed by inositol 1,3,4,5,6-pentaphosphate 2-kinase (OsIPK1). CRISPR/Cas9-mediated mutagenesis generated small deletions in the gene, producing knockout alleles. The *osipk1_1* mutant had reduced inositol triphosphate and phytic acid content, diminished ROS accumulation and oxidative damage and tolerated better salt and osmotic stresses (Jiang et al. 2020). Engineering the inositol pathway by gene editing can therefore improve tolerance to environmental stresses such as salinity or drought.

Salinity generates osmotic and oxidative stresses which are similar to the effects of water deprivation during drought (Yang and Guo 2018). Hybrid proline-rich proteins (HyPRP) are known as cell wall structural components which can influence responses to certain biotic and abiotic stresses including salinity and drought. Downregulation of the stress-responsive *SlHyPRP1* gene in tomato enhanced tolerance to salt, osmotic and oxidative stresses, suggesting that it is a negative regulator of stress responses (Li et al. 2016). Deletion of the PRD domain by multiplex CRISPR/Cas9 genome editing resulted in enhanced salt tolerance of the tomato mutant (Tran et al. 2021). On the other hand, removal of the 8CM domain by gene editing improved tolerance to moderate heat stress. Elimination of these domains could enhance tolerance to high osmotics at seedling stage (Tran et al. 2023). CRISPR/Cas9-mediated dual mutagenesis of *SlHyPRP1* and *SlDEA1* genes in tomato could alleviate ROS accumulation and improve tolerance to drought, salinity as well as to some pathogenic bacteria (Saikia et al. 2024). These results demonstrate that carefully designed multiplex gene editing can be used to engineer certain multidomain proteins such as HyPRPs and generate novel alleles which can confer stress tolerance to crops.

These reports demonstrate that salt tolerance is amenable to improve through carefully designed editing of important signalling or transcription factors, ion transporters or metabolic regulators. Agricultural utility of a strategy relying on gene editing has recently been demonstrated. The PUSA rice DST1 was generated by knocking out a stress response suppressor in a commercial Indian variety MTU1010, using CRISPR/Cas gene editing. Mutants had reduced stomatal density, improved tillering and yield combined with considerable salt and drought tolerance, confirmed in field conditions. PUSA rice DST1 was recently released for agricultural production showing that salt-tolerant commercial rice varieties can be generated by carefully designed gene editing (Priyadarshini 2025; ICAR-IIR 2025).

An interesting approach has recently been reported to develop salt-tolerant rice using multiplex gene editing. Sea Rice 86 (SR86) is an *indica*-type, ancient salt-tolerant variety, which has a number of traits which make it unsuitable for commercial production. Thirteen genes were selected for targeted mutagenesis employing three-step, multiplex CRISPR/Cas9 gene editing system, aiming to improve agricultural properties of SR86. Aims were to reduce plant height (Os*SD1*), change plant development (Os*OTUB1*), modify grain shape (Os*GS3, OsGW8, OsGS9*), increase grain number (*OsGN1a*), reduce awn length (*OsAN-2*), improve grain quality (*OsWx, OsqSH1, OsBADH2*), nitrogen use efficiency (*OsARE1*) and to weaken photoperiod sensitivity (*OsHD1*). Editing usually created small indels producing knockout mutants successfully for 9 genes. To improve plant architecture, expression of *OsIPA1* gene was enhanced by eliminating a miR156 binding site in the coding region. Phenotyping in controlled and field conditions revealed that the generated *SR86M* mutant retained salt tolerance of the parental SR86 line, and displayed agronomic traits comparable to modern cultivated rice varieties (Table 2, Hao et al. 2025). This example demonstrates the power of multiplex gene editing to engineer complex agronomic traits and combine them with stress tolerance of less domesticated varieties.

### Tolerance to heavy metals

Heavy metals, such as cadmium, manganese, copper, arsenic, or cesium, usually are in low concentrations in soil, but can accumulate to toxic degrees in certain regions exposed to mining, contamination by industrial activity or affected by accidents. Cadmium (Cd) affects photosynthesis, generates oxidative stress and reduces plant growth. NRAMP-type and low affinity cation transporters (LCT) mediate uptake of several heavy metals including Cd. CRISPR/Cas9-mediated mutagenesis of *OsLCT1* and *OsNramp5* generated single and double rice mutant lines with reduced accumulation of Cd in cadmium-containing paddy fields. While *oslct1* mutant could cope with Cd toxicity in lightly contaminated soils, one of the *osnramp5* mutants was able to reduce Cd uptake even in heavily contaminated soils (Table 2, Songmei et al. 2019). A more recent study revealed that the *osnramp5* knockout line is indeed tolerant to high Cd concentrations (Table 2, Tang et al. 2022). Mutagenesis of another heavy metal transporter *OsNramp1* by CRISPR/Cas9 editing led to reduced accumulation of various heavy metals including Cd, Pb, Mn and Ni. The mutant had altered ROS levels and antioxidant activity and displayed resistance against various pathogenic bacteria and fungi (Chu et al. 2022). The bifunctional nucleotidase/phosphatase protein SAL1 is a general negative regulator of stress responses (Wilson et al. 2009; Estavillo et al. 2011). The knockout *sal1* mutant was found to tolerate toxic concentrations of Cd better than wild-type plants through alleviating oxidative and ER stress (Xi et al. 2016). The Low Cadmium (*OsLCD*) gene controls Cd accumulation and toxicity in rice. The CRISPR/Cas9-generated *oslcd* mutant tolerated better and accumulated lower Cd than wild-type plants, resulting in reduced oxidative damage. Beneficial effect of this mutation on Cd accumulation could be observed in plants grown on cadmium-containing fields also without growth or yield penalty (Table 2, Chen et al. 2023).

Cesium (Cs) is present in soil usually in negligible concentrations. Nuclear accidents can however enhance radiocesium (^137^Cs^+^) content, representing a serious environmental and health hazard. Membrane-localized HAK1 transporters cannot discriminate between potassium and cesium, and mediate uptake of both ions. *OsHAK1* transporter gene has been mutated by CRISPR/Cas9 editing to produce rice mutants with poor Cs transport capacity. Cs uptake was considerably reduced in the mutants not only in hydroponics system but also on radioactive contaminated soils of the Fukushima region of Japan. While seeds of wild-type rice plants accumulated ^137^Cs^+^ to more than a hundred times of the permitted level, radioactive cesium in seeds of *oshak1* mutants was one to two magnitude lower. Biomass and seed yield of the *oshak1* mutants were comparable to wild-type plants in the field, suggesting negligible deficiency in potassium metabolism (Table 2, Nieves-Cordones et al. 2017). These results demonstrate engineering membrane transporters by gene editing can efficiently reduce uptake and accumulation of harmful heavy metals and radioisotopes even in highly contaminated soils.

### Cold tolerance

Low temperatures affect plant growth and development in various ways. Chilling temperatures above freezing reduce growth, impair photosynthesis, alter membrane rigidity and profoundly affect plant metabolism. Subzero temperatures lead to extracellular ice formation, and can destroy cell membranes and cellular structures, leading to cell death. Responses to cold are coordinated by a complex regulatory pathway which controls membrane stabilization, metabolic adjustments, modulation of enzyme activities and production of protective compounds and proteins. Cold signaling pathways include calcium (Ca^2+^) signals, reactive oxygen species (ROS), microRNAs (miRNAs), a particular set of transcription factors, such as C-repeat binding factors (CBFs), CAMTA, and MYB-type proteins, which modulate the activities of large gene sets (Kidokoro et al. 2022). CBF factors are key activators of cold response regulation, which is controlled by a signaling module composed of SnRK2-type kinase OST1, the transcription factor ICE1, and the repressors HOS1 and HAT1 (Dong et al. 2006; Kang et al. 2025). Gene regulatory networks which control responses to cold, drought, and salinity are partially overlapping, sharing common components which respond to dehydration or ROS accumulation (Kim et al. 2024). Trehalose accumulating rice plants were shown to display enhanced tolerance to drought, salinity, and cold stress, suggesting that this compound has a general protectant feature (Jang et al. 2003). Enhanced ABA sensitivity of various arabidopsis and rice mutants or transgenic lines improved tolerance not only to drought but also to low temperatures (Tian et al. 2015; Lenka et al. 2018; Zhang et al. 2024). miR408 was shown to promote tolerance to salt, cold and oxidative stress by regulating the expression of genes with antioxidant functions (Ma et al. 2015). Engineering fatty acid biosynthesis with fatty acid desaturase 2 (FAD2) increased linoleic acid content and enhanced tolerance to salt and cold stress (Dar et al. 2017). A recent rice study identified genes implicated in MAPK and ABA signaling which regulate cold tolerance through reprogramming photosynthesis and carbon assimilation in low temperatures (Ding et al. 2025). Cold acclimation is an adaptive mechanism which facilitates tolerance to freezing temperatures through modulation of CBF activities and epigenetic changes including chromatin modifications preparing plants to adapt to cold and tolerate low temperatures which otherwise are damaging (Barrero-Gil and Salinas 2018; Kidokoro et al. 2022; Tian et al. 2022). Cold-related gene editing targets can therefore include members of the CBF regulon, components of MAPK, ABA, and calcium signaling, genes implicated in lipid metabolism, biosynthesis of protective compounds such as sugars or sugar alcohols, antifreeze proteins, and antioxidants (Kumari et al. 2024). CRISPR/Cas9 mutagenesis of *HOS1* generated a knockout arabidopsis mutant with similar cold tolerance to the T-DNA insertion *hos1-3* line, demonstrating that engineering cold signaling pathways through genome editing is feasible (Dong et al. 2006; Shkryl et al. 2021).

Tropical, subtropical crops such as rice are sensitive to low temperatures, which can seriously affect yields in regions exposed to cold days or nights. Increased tolerance to chilling temperatures is therefore an important issue for rice breeders and biotechnologists (Li et al. 2022a, b). Cold tolerance could be enhanced in rice by targeting various transcription factors. OsMYB30 is implicated in stress responses and shown to influence cold tolerance. CRISPR/Cas9-mediated multiple editing of *OsPIN5b*, *GS3* and *OsMYB30* genes in rice could increase cold tolerance and improve yields in adverse conditions (Zeng et al. 2020). The transcription factor OsWRKY63 is a negative regulator of cold tolerance in rice and suppresses the activity of positive regulatory factors *OsWRKY76* and *OsDREB1B*, promoting cold response. CRISPR/Cas9-generated knockout *OsWRKY63* mutants displayed derepressed activity of these factors and were more tolerant to chilling temperatures, demonstrating that targeted mutagenesis of a single repressor can have considerable positive effect on cold tolerance (Zhang et al. 2022a, b). The NAC domain transcription factor OsNAC050 is another negative regulator of cold responses, which controls thousands of rice genes. CRISPR/Cas9-mediated mutagenesis of *OsNAC050* improved tolerance to low temperature treatments through modulating photosynthetic activity and sugar metabolism as well as reducing ROS accumulation and oxidative damage (Wang et al. 2023).

Besides rice, genome editing was adapted to other crops to mutagenize cold-related genes, including horticultural crops. Tomato is an important vegetable of tropical origin and is sensitive to low temperatures. SlNAC3 transcription factor is implicated in abiotic stress responses, including cold. Overexpression of *SlNAC3* increased cold sensitivity, while CRISPR/Cas9-derived *slnac3* mutants displayed cold tolerance in a CBF-independent way. *SlNAC3* was found to promote ethylene synthesis, which was considerably reduced in the mutant, facilitating better recovery and growth in chilling temperatures (Wang et al. 2024).

Starch degradation and sucrose cleavage in cold-exposed potato tubers are mediated by vacuolar acid invertase (Vlnv), producing hexoses for osmoprotection. Editing of *StVlnv* in two potato cultivars reduced sugar accumulation and led to increased antioxidant activities containing ROS-dependent oxidative damage in cold-treated tubers. Tubers of the *stvlnv* mutants were less damaged and could be longer maintained in low temperature storage conditions (Teper-Bamnolker et al. 2023). These data show that tolerance to low temperatures can be improved even in crops with tropical origin by well-designed mutagenesis of key regulatory genes.

### Plant organelles and stress responses

Plant chloroplasts and mitochondria derive from endosymbiotic bacteria housing essential cellular functions, such as photosynthesis and respiration. Both organelles are metabolic powerhouses and are implicated in physiological processes related to growth, development, in generation and scavenging of ROS, maintenance of redox balance in adverse conditions. Biosynthesis of certain amino acids, precursors of plant hormones and secondary metabolites takes also place in these organelles.

Chloroplasts are the sites of photosynthesis, which is one of the most sensitive processes to environmental stress. During salt and drought stress, photosynthesis is affected by stomata closure which limits gas exchange as well as by the oxidative stress generated by accelerated ROS production. Such conditions repress the activity of photosynthesis-related genes (Chaves et al. 2009). Non-photochemical quenching (NPQ) in photosystem II (PSII) is a conserved mechanism of photoprotection which can alleviate stress-imposed damage (Lu et al. 2022). Photosystem II Subunit S (PsbS) functions as a light sensor, controls photosynthetic energy conversion and is a key regulator of NPQ (Valencia and Pandit 2024). 5’ region of the *OsPSBS1* gene in rice was engineered by multiplex CRISPR/Cas9 to generate numerous indels and inversions, producing overexpression, knockout, or knock-down phenotypes. *OsPSBS1* gain-of function overexpressing mutants had higher NPQ, reduced stomatal conductance and better water use efficiency (WUE), important features for drought tolerance (Patel-Tupper et al. 2024). *psbA* gene is localized in the plastid DNA and encodes the chloroplast D1 protein, an essential component of PS II, which is susceptible to photodamage in stress conditions. Overexpression of maize *ZmpsbA* in tobacco stabilizes photosynthesis, conferring drought tolerance (Huo et al. 2016). Targeted point mutations in the *psbA* gene via TALED/DdCBE stabilized the D1 protein and conferred heat and high light tolerance in arabidopsis, tobacco, and lettuce (Mok et al. 2022, 2024). A point mutation in the same gene was also reported to render soybean tolerant to high temperatures and to the atrazine herbicide (Alfonso et al. 2001).

Chloroplasts are important ROS generators in plant cells, producing singlet oxygen, superoxide anion, hydrogen peroxide, and hydroxyl radicals (Mignolet-Spruyt et al. 2016). Abiotic stresses exacerbate ROS production, overpowering the intrinsic ROS-scavenging system generating oxidative stress (Zhu. 2016; Noctor et al. 2018). Hence, chloroplasts are important targets for gene editing to reduce ROS production and alleviate oxidative damage. The nuclear-encoded but chloroplast-localized HIGH CHLOROPHYLL FLUORESCENCE 106 (HCF106) and THYLAKOID FORMATION 1 (THF1) proteins form a complex and regulate ROS levels in guard cells. Hyperaccumulation of ROS in guard cells of single and double *hcf106* and *thf1* mutants resulted in faster stomata closure and better water retention in response to water deficiency (Wang et al. 2016). The PsbP Domain Protein 5 (PPD5) and the chloroplast protein THF1 were found to have related function*,* as the *ppd5* and *thf1-1* mutants hyperaccumulated H_2_O_2_ in guard cells, which promoted stomatal closure in water-stressed plants. The SnRK2-type protein kinase OST1 phosphorylates PPD5, which suppresses tolerance to water deficiency through OST1-dependent ROS signals (Hong et al. 2020). *PPD5* and *THF1* can therefore be targets of gene editing in crops to augment drought tolerance. The chloroplast calcium uniporter cMCU is implicated in stress-dependent calcium uptake into chloroplasts. The *cmcu-1* and *cmcu-2* insertion mutants had higher ABA content, showed faster stomata closure, and were able to maintain photosynthetic activity in water-restricted conditions. Large-scale alterations in protein abundances were found in *cmcu* mutants, many of them involved in calcium homeostasis, chlorophyll synthesis and retrograde signaling (Corti et al. 2023). Potassium (K^+^) has also important physiological functions in chloroplasts to maintain photosynthesis. Chloroplast K^+^Efflux Antiporters KEA1 and KEA2 in arabidopsis were shown to be essential to maintain K^+^ homeostasis in plastids. The double mutant *kea1,kea2* had altered ROS and NO levels associated with enhanced photorespiration leading to faster stomata closure, which led to increased tolerance to water deficiency and improved recovery after re-watering (Sánchez-McSweeney et al. 2021). Such reports suggest that a set of plastid-localized proteins can control peroxide levels in guard cells and regulate stomatal aperture. Modulation of the activity of such proteins by targeted mutagenesis can therefore be a promising strategy to engineer ROS signalling and improve drought and salt tolerance. Such a strategy was recently verified in poplar. CRISPR/Cas9-mediated genome editing introduced mutations in the promoter of *PagHCF106* gene in poplar (*Populus alba* × *Populus glandulosa*) creating knock-down mutants with reduced transcript levels. The generated mutants had enhanced peroxide levels in guard cells, displayed reduced stomatal opening, which led to better water retention in water-restricted conditions (Liu et al. 2025b).

Β-Carbonic anhydrases (βCAs) are localised in chloroplasts and are involved in uptake, fixation and recycling of CO_2_ during photosynthesis and modulate photosynthetic electron transport in different light conditions (Dąbrowska-Bronk et al. 2016; Rudenko et al. 2025). Nuclear and organellar transcript profiles of *βca1* and *βca2* arabidopsis mutants were dramatically altered, including many plastidic and mitochondrial genes. The *βca1* and *βca2* mutants were able to survive water depletion that proved lethal to wild-type plants. Their photosynthetic activity remained high during serious water stress and subsequent re-watering. Drought tolerance was suggested to derive from reduced and altered βCA1 and βCA2 activities that could modulate CO_2_-controlled stomatal movements in guard cells (Xu et al. 2023). Such metabolic changes likely reconfigure stress response pathways to enhance survival under water deficit.

Chloroplast-derived signals can influence expression of a set of nuclear-encoded genes implicated in light, hormonal and stress responses, through retrograde signaling (Loudya et al. 2024). The metabolite 3’-phosphoadenosine 5’-phosphate (PAP) functions as secondary messenger and retrograde signal during stress, regulating RNA and sulphur metabolism, ROS and ABA signals in stomatal closure (Estavillo et al. 2011; Pornsiriwong et al. 2017). The arabidopsis *SAL1/FIERY1* gene encodes a bifunctional enzyme with 3'(2'),5'-bisphosphate nucleotidase and inositol polyphosphate 1-phosphatase activities, is implicated in chloroplast to nucleus retrograde signaling, modulates ABA signals, regulates stomatal closure, seed germination, and controls miRNA and siRNA abundance and function. Mutants of SAL1 (*sal1/hos2/fry1/axl8*) constitutively accumulate PAP, are hypersensitive to ABA, display enhanced expression of stress-induced genes and are more tolerant to drought (Wilson et al. 2009; You et al. 2019). In guard cells, the SAL1-PAP pathway promotes ROS accumulation by enhancing RBOHD activity, subsequently upregulates calcium-dependent protein kinases (CPKs) and the slow ion channel SLAC1, needed for stomata closure (Tee et al. 2025). Engineering PAP-dependent retrograde signaling by genome editing of *SAL1* genes in crops is an attractive strategy to enhance drought tolerance. Enhanced PAP content in *sal1* mutants, however, generates growth and developmental defects which should be reduced in biotechnology applications (Phua et al. 2018). Multiplex CRISPR/Cas9 system was recently used to mutagenize five *TaSal1* genes in hexaploidy wheat, to engineer the PAP-controlled tolerance traits in this important crop with complex genomes. The wheat mutants had rolled leaf phenotype with closed stomata and were more tolerant to PEG-provoked dehydration (Table 1, Mohr et al. 2022; Abdallah et al. 2025). These results demonstrate the power of multiplex gene editing in plants with complex genomes and show that SAL1/PAP-controlled signals can modulate drought tolerance not only in model plants but also in important cereals as well.

Mitochondrial respiration is an important energy source for plants in non-photosynthetic tissues and in the absence of light. The mitochondrial electron transport chain (mETC), is the central pathway for oxidative phosphorylation generating ATP through oxidation of electron donors such as NADH or ascorbate (Møller 2001). Mitochondria host numerous metabolic processes, control photorespiration, stabilize cellular redox balance, regulate programmed cell death and modulate pathogen defenses (Møller et al. 2021). In plants, mETC can be bypassed by alternative oxidases (AOX) and type II NAD(P)H dehydrogenases (NDs), which can contribute to maintain electron flow, alleviate over-reduction of mETC and reduce ROS accumulation in stress conditions (Noctor et al. 2018; Van Aken 2021; Møller et al. 2021). NDs and AOX can alleviate damages and increase tolerance to drought, salinity and other stresses (Sweetman et al. 2019; Vanlerberghe et al. 2020; Oh et al. 2022). While most of the mitochondrial proteins are encoded in the nuclear genome, around 10% of the mitochondrial protein-coding genes are in the mitochondrial DNA which is a highly dynamic and heterogeneous subgenome (Gualberto and Newton 2017; van Wijk et al. 2024).

PPR proteins are targeted either to chloroplasts or mitochondria, where many of them are involved in processing of organellar RNAs (Barkan and Small 2014). AHG11 is a mitochondrial PPR-type protein, needed for editing of *nad4* transcript. The *ahg11* mutant is hypersensitive to ABA and characterized by enhanced ROS levels (Murayama et al. 2012). The Mitochondrial Single-Stranded DNA-Binding Protein (SSB1) is a nucleus-encoded mitochondrial ssDNA-binding protein, which is involved in splicing of nad1 and nad2 RNAs. The *ssb1-1* mutant was found to accumulate higher amounts of ROS and is hypersensitive to ABA (Qian et al. 2021). *ABO5* is an ABA hypersensitive mutant with enhanced ROS levels, high expression of *AOX1a,* but reduced induction of stress and photosynthesis-related genes. The PPR protein ABO5 is needed for splicing of transcripts of *nad2*, a subunit of complex I (Liu et al. 2010). ABA hypersensitivity was reported for other mutants encoding dysfunctional splicing proteins, such as DEXH box RNA helicase ABO6 (He et al. 2012), RCC1 family protein RUG3 (Kühn et al. 2011), and mitochondrial CRM protein CFM9 (Lee et al. 2019). ABA hypersensitivity of most these mutants was, however, demonstrated in vitro conditions and their ABA and drought-related phenotype in soil-grown plants is not known. One of the few examples is the arabidopsis *SLO2* mutant, which had reduced mETC rates, enhanced AOX activity, ABA hypersensitivity and ethylene insensitivity leading to enhanced tolerance to salinity and drought. *SLO2* encodes a mitochondrial pentatricopeptide repeat (PPR) protein involved in RNA editing of mitochondrial transcripts (Zhu et al. 2014).

Several mutants disrupting or affecting the mETC can influence responses to abiotic stress. The arabidopsis *ndufs4* mutant lacks the NADH dehydrogenase [ubiquinone] fragment S subunit 4 (NDUFS4), an essential component of mETC complex I. Mutant seedlings show increased tolerance to cold, mild salt, and osmotic stress, and improved survival after water deprivation (Meyer et al. 2009). Inactivation of the *NDUFS8.2* gene partially inhibited oxygen consumption through complex I. The *ndufs8.2–1* mutant had enhanced tolerance to water deficiency, which could be the consequence of the activated ND and AOX pathways (Zsigmond et al. 2024). PPR40 is associated with mETC complex III and is important to maintain the ubiquinol–cytochrome c oxidoreductase activity. The ABA hypersensitive *ppr40-1* mutant has enhanced ROS accumulation, oxidative damage and expression of the *AOX1a* gene (Zsigmond et al. 2008). Soil-grown *ppr40-1* displayed improved survival rates of drought-exposed plants (Kant et al. 2024). These reports suggest that mitochondrial functions linked to complexes I and III of mETC can be potential targets for modification in crops through editing the encoding nuclear genes.

Besides mETC, other mitochondrial functions can also be targeted to engineer stress responses. The Suppressor of hot1-4 (SHOT1) encodes a mitochondrial transcription termination factor (mTERF)-type protein, implicated in retrograde signaling and antioxidant activities. *shot1-1* and *shot1-2* mutants displayed enhanced tolerance to high temperatures, which could derive from contained oxidative damage (Kim et al. 2012, 2021). A mutation which disrupts the CMSII respiratory complex I in *Nicotiana sylvestris* had reduced respiration and increased antioxidant activity. Lower stomatal conductance of the mutant delayed water loss and increased viability in water deficiency (Djebbar et al. 2012). The arabidopsis AAA ATPase cytochrome bc1 synthase 1 (BCS1) participates in mETC stabilization, SA-dependent cell death, and pathogen defenses (Zhang et al. 2014). OsAAA-1 and OsAAA-2 ATPases in rice were found to be negative regulators of stress responses. CRISPR/Cas9-mediated mutagenesis of *OsAAA-1* produced knockout lines with higher seed yield and drought tolerance confirmed also in field conditions (Table 2, Lu et al. 2020). These results demonstrate that engineering mitochondrial traits by genome editing is a feasible strategy to improve drought and salt tolerance in crops.

### Plant development and stress responses

Plant development and architecture are controlled by a number of genes and influence responses to abiotic stresses, such as drought and salinity (Rahmati Ishka and Julkowska 2023). Most of the developmental changes during water deprivation correspond to avoidance mechanisms, and not tolerance at the cellular level. Mutant alleles of the Salt Root:shoot Ratio Regulator Gene (*SR3G*) in arabidopsis were identified by GWAS analysis and were found to be tolerant to salt stress. SR3G controls root: shoot ratio via root suberisation, root elongation and shoot growth, and is considered to be a negative regulator of salt tolerance (Rahmati Ishka et al. 2025). Engineering of root structure could improve drought tolerance in a few crops also. loss-of-function mutations of the wheat 12-OXOPHYTODIENOATE REDUCTASE (*OPRIII*) gene enzyme were shown to alter root architecture and enhance water uptake from soil through modulating jasmonate metabolism and ROS distribution (Gabay et al. 2023). Formation and growth of lateral roots in maize were shown to be regulated by *ZmLRT*, encoding the microRNA miR166a. While overexpression reduced lateral root number, gene-edited knockouts had more lateral roots. ZmLRT repressed the transcript levels of class III homeodomain-leucine zipper factors, implicated in root development. The gene-edited maize mutant displayed tolerance to water-restricted conditions, confirming that the lateral root system is important for water uptake in drought conditions (Zhang et al. 2023). The Grain number, plant height, and heading date2 gene in rice (*OsGhd2*) encodes a CCT transcription factor, which was found to positively control senescence in water-restricted conditions. Overexpression of *OsGhd2* accelerated senescence in plants exposed to water deprivation; the CRISPR/Cas9-generated knockout mutants displayed delayed senescence in such conditions (Liu et al. 2016). Orthologs of *Ghd2* can therefore be engineered in crops to contain senescence during drought. LATERAL ORGAN BOUNDARIES DOMAIN (LBD) transcription factors are known to regulate plant development. SlLBD40 of tomato is induced by and regulates responses to jasmonic acid (JA). Knockout *slbd40* mutants had reduced stomatal conductance and wilting, could retain water better than wild-type plants, had lower lipid peroxidation rates and higher photosynthetic activity in water-restricted conditions. Through modulation of JA signaling, SlLBD40 is a negative regulator of stress responses and its targeted mutagenesis could improve drought tolerance of tomato (Liu et al. 2020b). SQUAMOSA Promoter-Binding Protein-Like (SPL) genes encode transcription regulators which control various aspects of plant development and gibberellin signaling (Chen et al. 2010). The MsSPL8 factor regulates shoot branching in alfalfa and coordinates responses to water deficit. CRISPR/Cas9 gene editing produced small indels in *MsSPL8* resulting in morphological alterations as well as improved tolerance to water deficit (Singer et al. 2022). Alterations in growth habit can therefore have a consequence on drought tolerance.

Regulation of flowering is a critical aspect of plant development and is controlled by a complicated gene network. The arabidopsis SOC1/AGL20 and FUL/AGL8 proteins are MADS box transcription factors which are implicated in flowering control. The double *soc1,f*ul mutant was found to have reduced stomatal conductance, reduced leaf water potential and higher chlorophyll content in plants exposed to water deprivation. Reduced expression of most drought-induced genes suggested better water retention in dehydrating plants (Thonglim et al. 2023). Genes belonging to Flowering Locus T (FT) family are key regulators of flower initiation. gene-edited rice mutants of *OsFTL4* had early flowering phenotype, and were found to show improved tolerance to water deprivation in controlled conditions. Reduced stomatal conductance and water loss in water-restricted conditions alleviated drought-related damage leading to higher survival rates of the mutants (Gu et al. 2022). These reports show that regulatory genes implicated in flowering can have pleiotropic function and interfere with stress avoidance and tolerance.

Leaf rolling is a fast morphological response of plants to water deficiency which influences gas exchange, water retention, photosynthesis, carbon fixation and through that drought tolerance. In rice, leaf rolling is controlled by SEMI-ROLLED LEAF1 and SEMI-ROLLED LEAF2 (*OsSRL1*, *OsSRL2*) genes encoding putative glycosylphosphatidylinositol-anchored proteins. *srl1* and *srl2* mutants were generated by CRISPR/Cas9 tools and were found to have reduced stomatal conductance and transpiration rates. In water-restricted conditions, these mutants had higher ABA content, enhanced ROS-scavenging and reduced lipid peroxidation rates leading to higher survival rates after stress recovery (Liao et al. 2019). Xylan and lignin content can influence leaf morphology and responses to drought. Arabidopsis plants engineered for reduced lignin and xylan accumulation were found to withstand drought at higher frequencies (Yan et al. 2018). Arabidopsis *ixr* mutants with impaired xylan biosynthesis had delayed growth inhibition and higher survival rates in water-deficient conditions (Barbut et al. 2024). These data demonstrate that genes controlling leaf structure can be promising targets for editing for improvement of stress tolerance in crops.

### Chromatin and epigenetic regulation

Environmental stress generates large-scale changes in gene expression profiles which are influenced by alterations of chromatin structure, recognized as a key component of gene expression control. Epigenetic adjustments are reversible changes in plant genome, which include DNA methylation, generation of small RNAs and histone modifications which facilitate or prevent transcriptional activity (Matzke and Mosher 2014). DNA methylation is important for gene expression control and genome stability. In promoters, DNA methylation usually represses gene expression, but can enhance it when it happens in the gene body. DNA methylation patterns can be modified by abiotic or biotic stresses and influence gene activities (Zhang et al. 2018; Arora et al. 2022). Histone modifications include specific methylation, acetylation, phosphorylation and SUMOylation, which can modify chromatin compactness and accessibility of genes to transcription machinery. Genome-wide stress-dependent alterations in histone modification patterns have been described in several plant species, which were associated with the activities of defense-related gene sets. Such control modulates the activity of a large number of stress-regulated genes and contributes to stress adaptation (Thiebaut et al. 2019, Nunez-Vazquez et al. 2022). Epigenetic mechanisms have been recognized to facilitate stress adaptation through transmission of stress-generated epigenetic marks to progenies and transmit stress memory to offspring (Lämke and Bäurle 2017; Arora et al. 2022).

In plants, histone acetylation or particular methylation *H3K4me3* and *H3K36me2* are considered as activation marks, while *H3K27me3* is associated with silencing. Such epigenetic changes have an important function in the adaptation to extreme environmental conditions (Yu et al. 2025). In rapeseed, enhancement of H3K4me3 activation marks on loci activated by PEG-induced osmotic stress was associated with activation of a set of genes associated with stress response (Prasad et al. 2025). Modulation of histone methylation patterns by targeted mutagenesis of histone methyltransferases and/or demethylases can therefore be a promising strategy to modulate histone methylation patterns associated with stress tolerance. The rice Jumonji C domain-containing protein (OsJMJ710) has an H3K36me2 demethylase activity which plays an important role in stress-dependent histone modification. Mutagenesis of *OsJMJ710* by CRISPR/Cas9 gene editing led to enhanced H3K36 methylation levels on chromatin of the stress-related genes including the transcription factor *OsMYB48-1* gene, a positive regulator of drought tolerance. Upregulation of *OsMYB48-1* and other stress-related genes could enhance tolerance of the *OsJMJ710* mutant exposed to dehydration in controlled conditions (Zhao et al. 2022). A similar strategy was recently reported by creating knockout mutations in the JmjC domain rice gene Drought Tolerance 2 (DT2) with CRISPR/Cas9 technology. H3K9me2 methylation of the *OsZIP26* transcription factor gene was enhanced in the *osdt2* mutant, leading to reduced expression of it which alleviated down-regulation of *NCED2*, the key gene in ABA biosynthesis. Higher ABA content in the *osdt2* mutant promoted tolerance to water deficit manifested in significantly improved recovery of PEG-treated plants in growth chambers. Targeting *OsbZIP26* with CRISPR/Cas9 genome editing also resulted in drought-tolerant phenotype, similar to the *osdt2* mutant (Wang et al. 2025). These results revealed that engineering histone modifications by targeting specific histone demethylases can modulate expression of important stress regulators and promote adaptation to extreme conditions such as drought.

The SWI/SWF chromatin remodeling factor PICKLE (PKL) regulates chromatin structure and nucleosome density through histone modifications (Liang et al. 2024). PKL acts together with ABA and GA signaling to repress the activity of embryo-specific genes including *ABI3* and *ABI5* transcription factors and other regulatory genes such as At14a-Like 1 (*AFL1*), implicated in responses to drought (Perruc et al. 2007; Jing et al. 2023). *AFL1* encodes a membrane-associated protein related to b1-integrins, which can enhance growth and promote proline accumulation in water-limited environment (Kumar et al. 2015). PKL facilitates enrichment of the repressive H3K27me3 mark at the *AFL1* locus, thus preventing its transcription (Jing et al. 2023; Liang et al. 2024). In the absence of such repressive histone modification, *AFL1* is derepressed, conferring tolerance of the soil-grown *pkl* mutant to dehydration in controlled conditions (Jing et al. 2023). The evolutionarily conserved Elongator complex controls histone acetylation, transcription elongation, tRNA modification, secretion and is implicated in plant growth, development, and hormone responses including ABA and ROS signaling (Nelissen et al. 2010; Woloszynska et al. 2016). The largest subunit of the Elongator complex ELO2 is disrupted in the ABA-overly sensitive mutant *abo1,* which is hypersensitive to ABA in seedling growth and stomatal closure (Chen et al. 2006). Mutants of other Elongator subunits were also hypersensitive to ABA, resisted oxidative stress and had high anthocyanin accumulation (Zhou et al. 2009).

MicroRNAs are important regulators of DNA methylation and gene expression and silencing. Many studies demonstrate that miRNA can control stress responses in model and crop plants. In rice, OsmiR535 responds to drought and heavy metals. CRISPR/Cas9 genome editing generated knockouts of *OsmiR535*, which tolerated osmotic and salt stress as well as dehydration (Yue et al. 2020). *OsmiR535* mutagenesis allowed the activation of its target, the SPL7 transcription factor and subsequent suppression of *Nramp5* metal transporter. Cd accumulation was reduced in *OsmiR535* edited lines leading to considerable tolerance to this toxic metal as tested in controlled hydroponic conditions (Yue et al. 2023). The same OsmiR535 targets other SPL-type transcription factors, which control plant development or disease resistance (Zhang et al. 2022a, b). These results show that editing selected miRNA genes can modulate the activity of target genes implicated in tolerance to different stress conditions.

## Perspectives for genome editing in stress-related applications

**Fig. 2:**
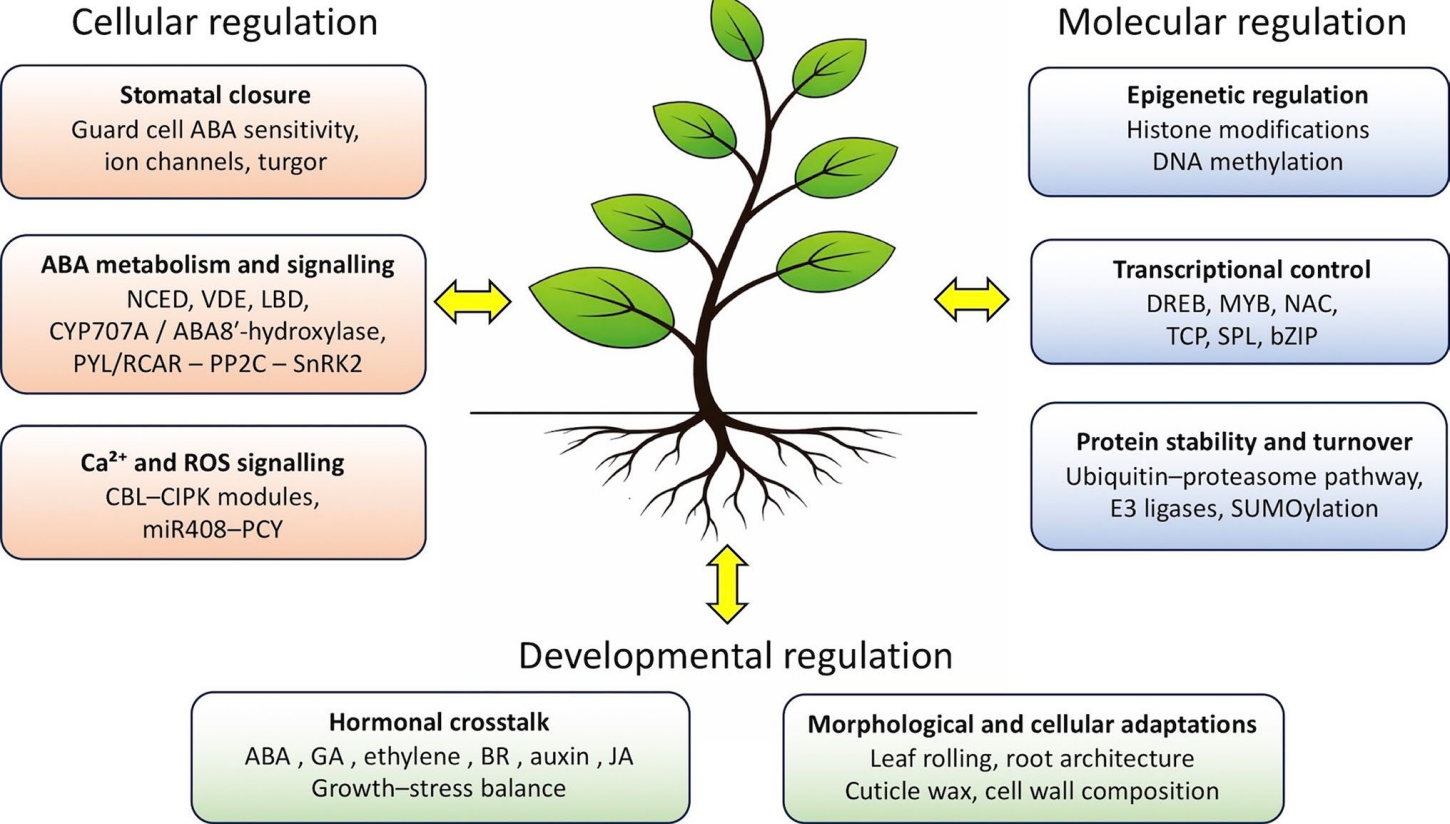
Possibilities of gene editing target procedures to improve tolerance to environmental stress conditions. Targets on cellular level influence rapid responses to environmental stimuli, and include various cellular signaling pathways. Responses on molecular level involve changes in gene activities, targeting epigenetic, transcriptional and protein regulation. Traits which modulate developmental and morphological features facilitate stress adaptation

Responses to extreme environmental conditions are typically multigene-controlled quantitative traits. Single mutations or gene modifications often generate minor changes which do not have significant effect on stress tolerance. There are however examples when genes encoding important regulatory or protective proteins could be engineered with success to achieve measurable degree of stress tolerance (Bowerman et al. 2023). Identification of target genes depends on high-quality genome sequences, which are available now for all important crops. Information derived from marker-assisted breeding, characterization of natural variability, mapping of SNPs and other genomic polymorphisms can be valuable to identify genes with potential for gene editing. Multiple processes can be considered for modification which influence responses to environmental effects. At a cellular level, manipulation of stomatal closure through engineering ABA levels or signaling, or altering ROS and Ca^2+^ signals can control water loss during dehydration. At a molecular level, editing key transcription or epigenetic factors modulates gene activities and transcript profiles, while protein abundances are influenced by translation and proteasome-dependent degradation. Developmentally controlled processes may contribute to stress avoidance through reducing evaporation or protecting photosynthesis. Plant hormones control many aspects of stress response, having enormous influence on tolerance (Fig. 2).

A straightforward approach is the CRISPR/Cas9-mediated mutagenesis of a characterized regulatory gene which downregulates stress responses, to achieve derepression of positive regulators, genes with protective function. A number of reports indicate that knocking out of a negative factor can improve tolerance to different abiotic stresses (Table S2). Recent advances in base or prime editing and homology directed repair (HDR) however allows the design of more complex genome modifications, including generation of gain-of function mutant alleles. Positive regulators of stress responses such as transcription factors, signaling proteins, antioxidants, or ion transporters can therefore be modified to boost their action (Bowerman et al. 2023; Kim et al. 2024). Such gain-of function phenotypes could be generated by editing *ZmARGOS8* in maize, *BnaA6.RGA* in rapeseed or *OsPYL9* in rice creating overexpression lines to improve drought tolerance, or *OsPSBS1* to enhance photosynthesis in rice (Shi et al. 2017; Wu et al. 2020; Usman et al. 2020; Patel-Tupper et al. 2024). These examples point to new avenues for gene editing, opening the spectrum of mutations to generate carefully designed phenotypes. Engineering energy supplying organellar functions associated with chloroplasts and mitochondria such as electron transport or redox balance offer particular perspectives to stabilize photosynthesis or modify respiration to alleviate negative effects of adverse conditions. Potential targets for gene editing are summarized in Fig. 2. and reviewed by Bowerman et al. (2023).

While such interventions can significantly improve stress tolerance, they may also incur fitness costs under non-stress conditions, such as dwarfism, developmental defects, or compromised fertility and yield. gene editing that substantially increases ABA levels or strongly sensitizes plants to ABA frequently shows developmental trade-offs. Examples include mutagenesis of rice *OsABA8ox2* and *OsVDE* or wheat *CYP707A* genes which increased ABA accumulation (Zhang et al. 2020; Wang et al. 2021b; Li et al. 2025), enhancement of ABA sensitivity of rapeseed through mutagenesis of the *BnaA6.RGA* gene (Wu et al. 2020), or multiplex editing of *TaSAL1* genes in wheat, which increased ABA sensitivity and drought tolerance (Abdallah et al. 2025). Such modifications improved drought or salt tolerance in various crops but had limited importance for commercial applications, which require a combination of valuable agronomic with stress tolerance. Targeting modifications to particular tissues, cells, or conditions may reduce such disadvantageous effects. Some of the gene editing efforts could, however, increase tolerance to environmental stresses with little or no detectable impact on growth or yield. Drought tolerance could be enhanced by CRISPR/Cas9 mutagenesis of the rice *OsDST* (Huang et al. 2009; Santosh-Kumar et al. 2020), down-regulation of rapeseed *BnFTA* (Wang et al. 2009), and tolerance to heavy metals could be improved by mutagenesis of *OsHAK1*, *OsNramp5, *or Os*LCD* genes in rice (Nieves-Cordones et al. 2017; Songmei et al. 2019; Chen et al. 2023) without compromising growth and yield. The recent release of the gene-edited KAMALA rice variety with drought tolerance and high yield indicates that genome editing can have great potential to cope with environmental constraints in changing climate (Solanki et al. 2026).

Responses to extreme environmental conditions are influenced by several genes and abiotic stress tolerance is a typical multigene-encoded character. To engineer such a complex trait, a number of genes should be simultaneously targeted. Multiplex gene editing is a technology of choice for such task and has a great potential to mutagenize a number of genes implicated in stress tolerance and adaptation. Several members of a gene family or different factors of a regulatory pathway can be targeted in such strategy. Editing of the *AITR* gene family in arabidopsis, tobacco, and soybean, or mutagenesis of the five *TaSAL1* genes in wheat enhanced drought or salt tolerance, maintaining growth and development comparable to wild-type plants (Chen et al. 2019, 2021a, b; Li et al. 2022a, b; Wang et al. 2021a; Table 1.). Alternatively, agronomic traits of a weakly domesticated genotype with strong stress tolerance can be modified by multiplex genome editing to combine desirable characters in an elite line. A number of ancient accessions and varieties are known to carry desirable tolerance traits which have already been lost in commercial varieties. Agronomic characters of such varieties can be improved by systematic editing of selected genes which determine quality, yield, or desirable growth parameters. Information on genome sequence is needed to design a multiplex editing program to generate elite lines with the tolerance trait. Feasibility of such strategy has recently been demonstrated by Hao et al. (2025), who were able to improve agronomic parameters of the ancient but salt-tolerant rice variety SR86, by simultaneously editing 9 genes. Such strategy can accelerate domestication of rural or ancient varieties, preserving the inherent tolerance trait and combine it with superior agronomic characters needed for agricultural production.

As genome editing creates genetic alterations similar to natural variations, registration of genome-edited crops can be less complicated and commercialization of gene-edited varieties easier with better public acceptance than of GM crops (Wang and Doudna 2023; Tuncel et al. 2025).

## Commercial possibilities

Scientific and technical advances in genetic technologies offer possibilities to improve tolerance of crop plants to extreme environmental conditions, which can be exploited only in proper legal and economic environment. In the absence of worldwide-accepted legislation, commercialization of GM and gene-edited crops is regulated on national level. There are two basic policies to handle regulatory issues. Product-based approaches focus on the qualities of the crop to be produced, regardless of the breeding technology used. Process-based regulation, however, puts emphasis on the engineering tools employed, requiring strict evaluation of the end product if it was generated by genetic intervention. These contrasting policies have a huge impact on the acceptance and production of GM crops and influence the commercial perspectives of gene-edited plants as well (Tachikawa and Matsuo 2024; Balyan et al. 2026). Today, GM crops are cultivated on 216 million hectares worldwide and have contributed to yield increases and stability allowing the adoption of more efficient agricultural practices, reducing losses to pests and pathogens, improving quality and tolerance to adverse conditions. Countries with the highest GM crop areas are USA, Brazil, Argentina, Canada, India and China, producing more than 90% of all engineered plants (AgbioInvestor 2026; Li et al. 2026). These countries have product-based GM regulation, facilitating commercial success of these varieties. Production of GM crops in most European countries is however low, mostly due to the prevailing process-dependent regulation and low public acceptance of genetic modifications, which can hamper commercialisation and cultivation of gene-edited crops as well (Friedrichs et al. 2022; Tachikawa and Matsuo 2024). Recent shifts in EU policy, however, indicate that such a situation might change. Adoption of new rules by the European Commission allows plants generated by New Genomic Techniques (particularly NGT1 category) to be treated similarly to varieties produced by conventional breeding. NGT1 category includes most applications of genome editing which carry small targeted genome modifications (less than 20 nucleotides) without traces of foreign DNA (EFSA 2025; Balyan et al. 2026). Recent regulatory changes can therefore open new opportunities for commercial genome editing in Europe, to develop crops with improved resilience to drought, salinity and other environmental stress conditions (Kovak et al. 2022; Adane and Alamnie 2024). Changing EU practices will likely have an influence on policies of other regions also. New trends in the legal environment lay the ground for innovative agricultural biotechnological companies such as Hudson River Biotechnology (https://www.hudsonriver.bio) which offer commercial services for DNA-free genome editing for a number of crops. Adjusted GM and NGT policies will likely promote the development of new, biotechnology-based breeding strategies to develop resilient crops for changing climate (Kovak et al. 2022; Tachikawa & Matsuo 2024; Balyan et al. 2026; Li et al. 2026).

Commercialization of gene-edited crops and vegetables has already begun but the number of examples is still low. Current developments are summarized in New GMOs Market Report 2025 (https://www.enga.org/fileadmin/user_upload/New_GMOs_Market-report-2025.pdf). A few examples illustrate the progress. TALEN technology was used by the company Calyx to generate a soybean variety with high oleic acid (Waltz 2019). CRISPR/Cas9-edited tomatoes enriched in γ-aminobutyric acid (GABA) have been released and sold by Sanatech Seed (Japan) (Waltz 2021). Maize lines resistant to Maize Lethal Necrosis (MLN) virus have been generated by editing the *eI4E* gene and are being released in several African countries (CYMMIT 2025). Reports for commercialization of gene-edited varieties with improved abiotic stress tolerance are still scarce. Research programs which could validate the superior tolerance character in field conditions can have a good chance to get close to commercial release of the improved varieties (Table 2). Two genome-edited rice varieties with improved drought or salt tolerance have recently been released by ICAR (Indian Council of Agricultural Research) and IIRR (Indian Institute of Rice Research) in India. The PUSA Rice DST1 has superior salt and drought tolerance, while the Kamal variety combines high yield and superior agronomic performance with drought tolerance (Priyadarshini 2025; ICAR-IIR 2025; Solanki et al. 2026). Such developments illustrate that new genomic technologies including gene editing will likely achieve a breakthrough in plant breeding and will be able to produce new, improved varieties with tolerance to extreme environmental conditions.

## Conclusions

Most reports on gene editing in relation with abiotic stresses have been published on arabidopsis and rice, followed by tomato, maize and soybean. Another dozen species were studied in a few papers. Most reports focused on drought, salt and cold tolerance, only a few studies included research on heavy metals, heat stress, or multiple stresses (Nascimento et al. 2023; Yadav et al. 2023). A number of metabolic and regulatory pathways were targeted in these studies to modulate hormonal metabolism and signaling, stress signal perception and transduction, to engineer various metabolic pathways including antioxidants, anthocyanins, lignin, proline, and oligosaccharides. A few reports described chloroplast and mitochondria-related traits such as photosynthesis or respiration (see supplementary data).

Complex traits such as drought tolerance can be addressed by combination of approaches targeting avoidance (eg. developmental characters), tolerance (eg. metabolic adjustments) and recovery mechanisms (recovery of homeostasis), essential to survive drought periods (Fig. 1). Due to the multigenic nature of stress tolerance and adaptation, importance of multiplex gene editing is likely increase in stress-related applications (see Table 1). Simultaneous editing of a number of genes has great potential to accelerate multigene-dependent breeding, facilitating domestication of neglected genotypes with valuable tolerance traits. Identification and editing of key regulatory genes in crop plants will have potential importance in improving drought or salt tolerance. Recent commercial release of gene-edited salt or drought-tolerant rice varieties (ICAR-IIR 2025) demonstrate the potential of single-gene approach.

Most of the phenotypic alterations generated by genetic transformation or genome editing have only been demonstrated in controlled conditions, such as greenhouse, growth chambers, and often using hydroponic cultures or other artificial substrates. Validation of tolerance traits in open field is however an important issue which faces a number of technical obstacles. In field conditions, environmental conditions are not controlled, and the desirable trait can be masked by the inherent variability of the plant population. A number of studies revealed that changes observed in controlled environment are not displayed in field conditions due to the complex soil and climatic conditions (Mittler and Blumwald 2010). Standardized field phenotyping would be an important tool to validate the tolerance traits in less-controlled environment resembling real agricultural conditions. Authorization for field tests of GM plants is however complicated. Table 2 compiles results of 20 field studies, which confirmed the improved stress tolerance in agronomical conditions. These examples are encouraging and confirm the potential of gene editing to generate novel varieties with enhanced resilience to adverse environmental effects, which can have commercial potential in the near future.

## Supplementary Information

Below is the link to the electronic supplementary material.

Supplementary file1 (XLSX 48 KB)

## Data Availability

No datasets were generated or analyzed during the current study.
